# Supplementary material for: Psychological well-being in Europe after the outbreak of war in Ukraine
Source: Nat Commun. 2024 Feb 20;15:1202. doi: 10.1038/s41467-024-44693-6 (PMC10879508; doi:10.1038/s41467-024-44693-6)
Supplement: Supplementary file 1 — Supplementary Information [file 41467_2024_44693_MOESM1_ESM.pdf]

Supplementary Information

Psychological well-being in Europe after the outbreak of war in Ukraine

Scharbert et al.

Corresponding author: Julian Scharbert
Email: julian.scharbert@uni-muenster.de

Overview

1. Supplementary Tables 2

1.1. Results of main analyses 2

1.2. Results of supplementary analyses controlling for country-specific effects 8

1.3. Results of supplementary analyses including global data 18

1.4. Results of supplementary analyses with different time frames 22

1.5. Results of supplementary analyses including lagged effects of the salience 30

1.6. Results of supplementary analyses with the sub-facets of personality and well-being 32

1.7. Results of supplementary analyses with societal well-being as outcome 39

1.8. Results of analyses with Ukraine-related variables 44

1.9. Results of analyses with Bayesian estimator 45

1. Supplementary Tables

In this section, we present the results of the main analyses (1.1) as well as the supplementary analyses with country-specific effects (1.2), global data (1.3), different time frames (1.4), lagged effects of the salience (1.5), the sub-facets of personality and well-being (1.6), societal well-being as outcome (1.7), the Ukraine-related variables (1.8), and with a Bayesian estimator (1.9).

1.1. Results of main analyses

Supplementary Table 1. Descriptive statistics of overall sample and country-specific sub samples.

Country	N participants	N observations	N days	Mean observations per participant	Gender: female	Gender: male	Gender: other	Age mean	Age min	Age max
Total	1735	54851	63	31.6	1372	349	14	28.1	18	91
Total (Europe)	1341	44894	63	33.5	1079	252	10	25.7	18	91
Argentina	47	1492	55	31.7	36	11	0	29.2	18	51
Australia	43	1386	57	32.2	33	6	4	42.5	27	75
Bahrain	1	12	8	12	0	1	0	21	21	21
Belgium	7	125	44	17.9	5	2	0	30.9	24	41
Brazil	23	713	46	31	14	9	0	39.7	19	79
Bulgaria	1	61	27	61	1	0	0	19	19	19
Cameroon	1	101	28	101	0	1	0	30	30	30
Canada	2	36	11	18	2	0	0	26	20	32
Chile	1	59	23	59	1	0	0	28	28	28
China	7	200	41	28.6	5	2	0	20	19	22
Croatia	1	6	3	6	1	0	0	29	29	29
Czech Republic	1	87	28	87	1	0	0	29	29	29
Ethiopia	1	9	6	9	0	1	0	51	51	51
France	61	993	39	16.3	51	10	0	26.4	18	65
Georgia	56	605	12	10.8	49	7	0	35	18	66
Germany	219	8507	63	38.8	182	34	3	33.1	18	91
Iceland	1	6	5	6	0	0	1	21	21	21
India	3	49	20	16.3	1	2	0	34	29	40
Ireland	1	13	9	13	0	1	0	27	27	27

Supplementary Table 1. (continued)

Country	N participants	N observations	N days	Mean observations per participant	Gender: female	Gender: male	Gender: other	Age mean	Age min	Age max
Italy	659	21051	45	31.9	520	136	3	22.7	18	76
Jamaica	1	18	10	18	1	0	0	30	30	30
Jordan	1	19	6	19	1	0	0	25	25	25
Latvia	1	3	3	3	1	0	0	32	32	32
Mexico	12	338	58	28.2	6	6	0	40.3	18	68
Mozambique	1	2	2	2	1	0	0	31	31	31
Namibia	2	90	25	45	2	0	0	28.5	27	30
Netherlands	22	893	51	40.6	17	4	1	32.8	18	72
Nigeria	3	91	33	30.3	1	2	0	35.3	32	38
Peru	2	102	24	51	1	1	0	22.5	19	26
Poland	103	3520	61	34.2	88	15	0	29.8	18	70
South Africa	139	3517	40	25.3	105	34	0	39.3	20	78
South Sudan	1	6	5	6	1	0	0	42	42	42
Spain	2	34	22	17	1	1	0	26	23	29
Sweden	2	32	10	16	2	0	0	38.5	26	51
Switzerland	3	61	25	20.3	3	0	0	37	31	42
Thailand	23	443	37	19.3	16	7	0	29	21	41
Turkey	112	4024	47	35.9	85	27	0	29.1	19	62
Uganda	3	66	28	22	1	2	0	38.7	35	42
United Kingdom	145	5478	63	37.8	121	22	2	20.5	18	59
Uruguay	1	46	23	46	1	0	0	42	42	42
USA	15	395	61	26.3	13	2	0	35.1	18	79
Zambia	3	127	28	42.3	0	3	0	32	25	37
Zimbabwe	2	35	19	17.5	2	0	0	29.5	21	38

Notes. “N participants” is the number of participants included in the analyses. “N observations” is the total number of state measurements for these participants. “N days” corresponds to the number of days with state assessments of at least one participant. The first row “Total” displays the descriptive statistics of the overall global sample. The second row “Total (Europe)” displays the descriptive statistics of the overall European sample.

Supplementary Table 2. Coefficients and model fits of eight multilevel models representing different well-being trajectories.

	Model 1a				Model 1b			
Predictors	b	95%-CI	*t*	*p*	b	95%-CI	*t*	*p*
(Intercept)	0.003	-0.037; 0.042	0.137	0.891	-0.046	-0.089; -0.003	-2.105	0.035
Level					-0.171	-0.229; -0.112	-5.721	<0.001
AIC	43134.68				43018.31			

	Model 1c				Model 1d			
Predictors	b	95%-CI	*t*	*p*	b	95%-CI	*t*	*p*
(Intercept)	0.021	-0.030; 0.072	0.801	0.423	-0.094	-0.160; -0.028	-2.779	0.005
Level					-0.201	-0.271; -0.131	-5.646	<0.001
Post-event	-0.060	-0.140; 0.019	-1.485	0.137	0.093	-0.002; 0.187	1.923	0.055
AIC	42933.87				42791.85			

	Model 2a				Model 2b			
Predictors	b	95%-CI	*t*	*p*	b	95%-CI	*t*	*p*
(Intercept)	0.018	-0.024; 0.059	0.838	0.402	-0.075	-0.130; -0.021	-2.700	0.007
Time	-0.063	-0.115; -0.010	-2.339	0.019	0.056	-0.014; 0.125	1.562	0.118
Level					-0.205	-0.284; -0.125	-5.046	<0.001
AIC	42965.48				42845.10			

	Model 2c				Model 2d			
Predictors	b	95%-CI	*t*	*p*	b	95%-CI	*t*	*p*
(Intercept)	-0.022	-0.080; 0.037	-0.717	0.473	-0.090	-0.156; -0.023	-2.644	0.008
Level					-0.200	-0.278; -0.122	-5.040	<0.001
Pre-event	-0.198	-0.297; -0.099	-3.923	<0.001	0.005	-0.097; 0.107	0.096	0.923
Post-event	-0.008	-0.093; 0.078	-0.174	0.862	0.089	-0.006; 0.184	1.837	0.066
AIC	42880.06				42781.12			

Notes. N = 1,341. Displays fixed-effect coefficients of multilevel models. All statistical tests were two-sided. The outcome of all models was well-being. The degrees of freedom were >10,000 for all statistical tests. b = unstandardized regression weight; 95%-CI = 95% confidence interval around the estimate.

Supplementary Table 3. Coefficients of Model 2d including additional level-2 predictors.

	**Model 2d**				**Model 2d + Stability**			
Predictors	b	95%-CI	*t*	*p*	b	95%-CI	*t*	*p*
(Intercept)	-0.090	-0.156; -0.023	-2.644	0.008	-0.090	-0.154; -0.025	-2.714	0.007
Level	-0.200	-0.278; -0.122	-5.040	<0.001	-0.182	-0.260; -0.103	-4.525	<0.001
Pre-event	0.005	-0.097; 0.107	0.096	0.923	0.041	-0.061; 0.143	0.781	0.435
Post-event	0.089	-0.006; 0.184	1.837	0.066	0.121	0.029; 0.212	2.571	0.010
Stability					0.245	0.180; 0.311	7.338	<0.001
Level * Stability					-0.027	-0.102; 0.048	-0.698	0.485
Pre-event * Stability					-0.043	-0.139; 0.054	-0.867	0.386
Post-event * Stability					0.161	0.066; 0.255	3.320	0.001

	**Model 2d + Age**				**Model 2d + Gender**			
Predictors	b	95%-CI	*t*	*p*	b	95%-CI	*t*	*p*
(Intercept)	-0.095	-0.162; -0.028	-2.787	0.005	-0.116	-0.190; -0.042	-3.064	0.002
Level	-0.188	-0.266; -0.109	-4.693	<0.001	-0.209	-0.295; -0.123	-4.745	<0.001
Pre-event	0.024	-0.076; 0.124	0.466	0.641	-0.000	-0.112; 0.111	-0.008	0.994
Post-event	0.105	0.010; 0.201	2.157	0.031	0.075	-0.031; 0.180	1.391	0.164
SDV	0.002	-0.071; 0.074	0.041	0.967	0.125	-0.047; 0.298	1.425	0.154
Level * SDV	-0.022	-0.105; 0.061	-0.522	0.602	0.047	-0.156; 0.250	0.454	0.650
Pre-event * SDV	-0.123	-0.228; -0.018	-2.301	0.021	-0.040	-0.326; 0.246	-0.271	0.786
Post-event * SDV	0.071	-0.034; 0.175	1.325	0.185	0.080	-0.166; 0.326	0.636	0.525

	**Model 2d + Political attitude**				**Model 2d + Social status**			
Predictors	b	95%-CI	*t*	*p*	b	95%-CI	*t*	*p*
(Intercept)	-0.091	-0.158; -0.024	-2.673	0.008	-0.090	-0.156; -0.023	-2.647	0.008
Level	-0.206	-0.284; -0.128	-5.177	<0.001	-0.199	-0.277; -0.121	-4.983	<0.001
Pre-event	0.008	-0.094; 0.111	0.159	0.874	0.026	-0.079; 0.130	0.480	0.631
Post-event	0.089	-0.006; 0.184	1.841	0.066	0.108	0.014; 0.202	2.241	0.025
SDV	0.009	-0.059; 0.078	0.273	0.785	0.099	0.032; 0.167	2.875	0.004
Level * SDV	-0.052	-0.132; 0.028	-1.280	0.200	0.011	-0.069; 0.091	0.264	0.791
Pre-event * SDV	-0.001	-0.108; 0.105	-0.027	0.978	-0.017	-0.126; 0.092	-0.308	0.758
Post-event * SDV	0.033	-0.064; 0.129	0.663	0.507	0.083	-0.014; 0.179	1.678	0.093

Notes. N = 1,341. Displays fixed-effect coefficients of multilevel models. All statistical tests were two-sided. The outcome of all models was well-being. Degrees of freedom were >10,000 for all

statistical tests. b = unstandardized regression weight; 95%-CI = 95% confidence interval around the estimate; SDV = sociodemographic variable as indicated in the column head.

Supplementary Table 4. Predicting well-being by the salience of the war and additional level-2 predictors.

	Salience				Salience (WS/BS)			
Predictors	b	95%-CI	*t*	*p*	b	95%-CI	*t*	*p*
(Intercept)	-0.004	-0.044; 0.035	-0.216	0.829	-0.008	-0.048; 0.032	-0.377	0.706
Tweets	-0.070	-0.096; -0.044	-5.298	<0.001				
Tweets (WS)					-0.065	-0.092; -0.037	-4.559	<0.001
Tweets (BS)					-0.116	-0.207; -0.024	-2.475	0.013

	Salience + Stability				Salience (WS/BS) + Stability			
Predictors	b	95%-CI	*t*	*p*	b	95%-CI	*t*	*p*
(Intercept)	-0.004	-0.039; 0.031	-0.200	0.842	-0.006	-0.042; 0.030	-0.336	0.737
Tweets	-0.071	-0.097; -0.045	-5.343	<0.001				
Tweets (WS)					-0.067	-0.095; -0.038	-4.629	<0.001
Tweets (BS)					-0.101	-0.184; -0.018	-2.395	0.017
Stability	0.335	0.299; 0.370	18.615	<0.001	0.334	0.298; 0.370	18.214	<0.001
Tweets * Stability	-0.013	-0.037; 0.012	-1.030	0.303				
Tweets (WS) * Stability					-0.010	-0.037; 0.016	-0.747	0.455
Tweets (BS) * Stability					-0.038	-0.118; 0.043	-0.917	0.359

	Salience + Age				Salience (WS/BS) + Age			
Predictors	b	95%-CI	*t*	*p*	b	95%-CI	*t*	*p*
(Intercept)	-0.002	-0.041; 0.037	-0.092	0.926	-0.004	-0.044; 0.036	-0.194	0.846
Tweets	-0.069	-0.095; -0.042	-5.067	<0.001				
Tweets (WS)					-0.064	-0.092; -0.035	-4.412	<0.001
Tweets (BS)					-0.107	-0.199; -0.014	-2.265	0.024
SDV	0.082	0.042; 0.123	4.012	<0.001	0.089	0.045; 0.132	4.018	<0.001
Tweets * SDV	-0.009	-0.036; 0.017	-0.669	0.503				
Tweets (WS) * SDV					-0.012	-0.040; 0.017	-0.798	0.425
Tweets (BS) * SDV					0.021	-0.071; 0.114	0.454	0.650

Supplementary Table 4. (continued)

	Salience + Gender				Salience (WS/BS) + Gender			
Predictors	b	95%-CI	*t*	*p*	b	95%-CI	*t*	*p*
(Intercept)	-0.032	-0.075; 0.012	-1.435	0.151	-0.038	-0.082; 0.007	-1.654	0.098
Tweets	-0.068	-0.096; -0.039	-4.623	<0.001				
Tweets (WS)					-0.059	-0.089; -0.028	-3.756	<0.001
Tweets (BS)					-0.141	-0.241; -0.040	-2.750	0.006
SDV	0.150	0.049; 0.251	2.924	0.003	0.159	0.057; 0.262	3.049	0.002
Tweets * SDV	-0.018	-0.086; 0.051	-0.501	0.617				
Tweets (WS) * SDV					-0.031	-0.103; 0.042	-0.827	0.408
Tweets (BS) * SDV					0.109	-0.136; 0.354	0.871	0.384

	Salience + Political attitude				Salience (WS/BS) + Political attitude			
Predictors	b	95%-CI	*t*	*p*	b	95%-CI	*t*	*p*
(Intercept)	-0.005	-0.044; 0.035	-0.227	0.821	-0.007	-0.047; 0.033	-0.333	0.739
Tweets	-0.072	-0.098; -0.045	-5.347	<0.001				
Tweets (WS)					-0.066	-0.094; -0.039	-4.659	<0.001
Tweets (BS)					-0.109	-0.202; -0.016	-2.308	0.021
SDV	0.036	-0.003; 0.076	1.814	0.070	0.039	-0.002; 0.079	1.870	0.061
Tweets * SDV	-0.013	-0.040; 0.015	-0.910	0.363				
Tweets (WS) * SDV					-0.014	-0.044; 0.015	-0.954	0.340
Tweets (BS) * SDV					0.009	-0.088; 0.107	0.189	0.850

	Salience + Social status				Salience (WS/BS) + Social status			
Predictors	b	95%-CI	*t*	*p*	b	95%-CI	*t*	*p*
(Intercept)	-0.002	-0.040; 0.037	-0.079	0.937	-0.004	-0.043; 0.036	-0.186	0.852
Tweets	-0.070	-0.096; -0.044	-5.277	<0.001				
Tweets (WS)					-0.066	-0.094; -0.038	-4.634	<0.001
Tweets (BS)					-0.103	-0.194; -0.012	-2.221	0.026
SDV	0.148	0.110; 0.187	7.509	<0.001	0.150	0.110; 0.190	7.378	<0.001
Tweets * SDV	0.003	-0.024; 0.030	0.201	0.841				
Tweets (WS) * SDV					0.001	-0.028; 0.030	0.048	0.962
Tweets (BS) * SDV					0.030	-0.065; 0.125	0.620	0.535

Notes. N = 1,341. Displays fixed-effect coefficients of multilevel models. All statistical tests were two-sided. The outcome of all models was well-being. Degrees of freedom were >10,000 for all statistical tests. WS = within-subjects component; BS = between-subjects component;

b = unstandardized regression weight; 95%-CI = 95% confidence interval around the estimate; SDV = sociodemographic variable as indicated in the column head.

1.2. Results of supplementary analyses controlling for country-specific effects

Supplementary Table 5. Coefficients of Model 2d including country variables.

	Model 2d				Model 2d + Stability			
Predictors	b	95%-CI	*t*	*p*	b	95%-CI	*t*	*p*
(Intercept)	0.143	-0.190; 0.476	0.843	0.399	0.068	-0.233; 0.370	0.444	0.657
Level	-0.159	-0.238; -0.081	-3.983	<0.001	-0.160	-0.241; -0.079	-3.871	<0.001
Pre-event	0.071	-0.030; 0.173	1.379	0.168	0.086	-0.020; 0.191	1.596	0.110
Post-event	0.096	-0.002; 0.193	1.925	0.054	0.099	0.004; 0.194	2.052	0.040
Stability					0.235	0.168; 0.301	6.924	<0.001
Level * Stability					-0.035	-0.110; 0.041	-0.897	0.370
Pre-event * Stability					-0.050	-0.146; 0.047	-1.014	0.311
Post-event * Stability					0.148	0.054; 0.243	3.070	0.002
France	0.318	-0.071; 0.707	1.602	0.109	0.294	-0.060; 0.647	1.630	0.103
Germany	-0.000	-0.340; 0.340	-0.000	1.000	-0.080	-0.387; 0.227	-0.512	0.609
Italy	-0.349	-0.683; -0.015	-2.046	0.041	-0.201	-0.504; 0.101	-1.303	0.193
Netherlands	-0.131	-0.569; 0.308	-0.584	0.559	-0.132	-0.528; 0.263	-0.655	0.512
Poland	-0.231	-0.585; 0.123	-1.281	0.200	-0.146	-0.465; 0.172	-0.900	0.368
Turkey	-0.252	-0.604; 0.100	-1.402	0.161	-0.256	-0.573; 0.062	-1.577	0.115
United Kingdom	-0.092	-0.439; 0.255	-0.521	0.602	0.010	-0.303; 0.322	0.061	0.951

Supplementary Table 5. (continued)

	Model 2d + Age				Model 2d + Gender			
Predictors	b	95%-CI	*t*	*p*	b	95%-CI	*t*	*p*
(Intercept)	0.125	-0.207; 0.457	0.738	0.460	0.125	-0.217; 0.467	0.714	0.475
Level	-0.144	-0.224; -0.063	-3.491	<0.001	-0.168	-0.255; -0.080	-3.735	<0.001
Pre-event	0.083	-0.019; 0.186	1.593	0.111	0.071	-0.042; 0.184	1.226	0.220
Post-event	0.105	0.006; 0.204	2.086	0.037	0.080	-0.028; 0.188	1.459	0.145
SDV	-0.028	-0.103; 0.047	-0.744	0.457	0.130	-0.042; 0.303	1.478	0.139
Level * SDV	-0.054	-0.138; 0.030	-1.255	0.209	0.044	-0.158; 0.247	0.428	0.669
Pre-event * SDV	-0.115	-0.220; -0.010	-2.151	0.031	-0.056	-0.339; 0.226	-0.391	0.696
Post-event * SDV	0.066	-0.038; 0.171	1.244	0.213	0.102	-0.143; 0.347	0.815	0.415
France	0.324	-0.064; 0.713	1.638	0.101	0.314	-0.081; 0.709	1.560	0.119
Germany	-0.024	-0.363; 0.314	-0.141	0.888	0.003	-0.345; 0.350	0.016	0.987
Italy	-0.333	-0.667; 0.001	-1.956	0.051	-0.363	-0.705; -0.022	-2.086	0.037
Netherlands	-0.130	-0.567; 0.307	-0.583	0.560	-0.141	-0.589; 0.308	-0.614	0.539
Poland	-0.237	-0.590; 0.115	-1.321	0.187	-0.235	-0.596; 0.125	-1.279	0.201
Turkey	-0.240	-0.591; 0.111	-1.339	0.181	-0.269	-0.629; 0.090	-1.469	0.142
United Kingdom	-0.021	-0.368; 0.326	-0.119	0.906	-0.089	-0.443; 0.266	-0.490	0.624

Supplementary Table 5. (continued)

	Model 2d + Political attitude				Model 2d + Social status			
Predictors	b	95%-CI	*t*	*p*	b	95%-CI	*t*	*p*
(Intercept)	0.146	-0.186; 0.478	0.862	0.389	0.146	-0.185; 0.476	0.865	0.387
Level	-0.165	-0.245; -0.085	-4.044	<0.001	-0.166	-0.246; -0.086	-4.058	<0.001
Pre-event	0.072	-0.032; 0.176	1.365	0.172	0.078	-0.028; 0.185	1.438	0.150
Post-event	0.098	0.000; 0.196	1.970	0.049	0.110	0.013; 0.208	2.219	0.026
SDV	0.017	-0.051; 0.085	0.489	0.625	0.074	0.006; 0.143	2.123	0.034
Level * SDV	-0.044	-0.124; 0.035	-1.090	0.276	0.006	-0.073; 0.086	0.154	0.877
Pre-event * SDV	0.002	-0.103; 0.107	0.042	0.967	-0.015	-0.124; 0.094	-0.272	0.786
Post-event * SDV	0.033	-0.062; 0.129	0.685	0.493	0.091	-0.005; 0.187	1.850	0.064
France	0.312	-0.077; 0.700	1.572	0.116	0.289	-0.096; 0.674	1.472	0.141
Germany	-0.004	-0.343; 0.336	-0.021	0.983	-0.040	-0.377; 0.298	-0.231	0.818
Italy	-0.355	-0.689; -0.022	-2.090	0.037	-0.337	-0.668; -0.006	-1.993	0.046
Netherlands	-0.128	-0.565; 0.310	-0.572	0.568	-0.181	-0.616; 0.255	-0.813	0.416
Poland	-0.228	-0.581; 0.124	-1.269	0.204	-0.236	-0.587; 0.115	-1.319	0.187
Turkey	-0.257	-0.609; 0.094	-1.436	0.151	-0.262	-0.611; 0.087	-1.470	0.142
United Kingdom	-0.101	-0.447; 0.245	-0.572	0.567	-0.100	-0.443; 0.244	-0.568	0.570

Notes. N = 1,341. Displays fixed-effect coefficients of multilevel models. All statistical tests were two-sided. The outcome of all models was well-being. Degrees of freedom were >10,000 for all statistical tests. b = unstandardized regression weight; 95%-CI = 95% confidence interval around the estimate; SDV = sociodemographic variable as indicated in the column head.

Supplementary Table 6. Predicting well-being by the salience of the war, country, and additional level-2 predictors.

	Salience				Salience (WS/BS)			
Predictors	b	95%-CI	*t*	*p*	b	95%-CI	*t*	*p*
(Intercept)	0.228	-0.096; 0.553	1.382	0.167	0.248	-0.073; 0.569	1.512	0.131
Tweets	-0.068	-0.094; -0.042	-5.068	<0.001				
Tweets (WS)					-0.070	-0.098; -0.042	-4.931	<0.001
Tweets (BS)					-0.044	-0.137; 0.050	-0.909	0.363
France	0.288	-0.088; 0.663	1.500	0.134	0.271	-0.102; 0.644	1.423	0.155
Germany	-0.003	-0.341; 0.335	-0.018	0.985	-0.022	-0.341; 0.313	-0.127	0.899
Italy	-0.380	-0.708; -0.051	-2.267	0.023	-0.398	-0.724; -0.072	-2.396	0.017
Netherlands	-0.140	-0.574; 0.294	-0.631	0.528	-0.164	-0.599; 0.271	-0.739	0.460
Poland	-0.257	-0.609; 0.095	-1.430	0.153	-0.284	-0.633; 0.065	-1.593	0.111
Turkey	-0.260	-0.610; 0.090	-1.458	0.145	-0.286	-0.634; 0.062	-1.613	0.107
United Kingdom	-0.084	-0.429; 0.261	-0.479	0.632	-0.094	-0.436; 0.247	-0.541	0.588

	Salience + Stability				Salience (WS/BS) + Stability			
Predictors	b	95%-CI	*t*	*p*	b	95%-CI	*t*	*p*
(Intercept)	0.154	-0.141; 0.450	1.025	0.305	0.161	-0.131; 0.453	1.079	0.281
Tweets	-0.064	-0.091; -0.038	-4.816	<0.001				
Tweets (WS)					-0.068	-0.096; -0.040	-4.694	<0.001
Tweets (BS)					-0.032	-0.118; 0.054	-0.723	0.470
Stability	0.318	0.282; 0.354	17.279	<0.001	0.318	0.281; 0.355	16.949	<0.001
Tweets * Stability	-0.013	-0.038; 0.011	-1.067	0.286				
Tweets (WS) * Stability					-0.009	-0.036; 0.017	-0.703	0.482
Tweets (BS) * Stability					-0.044	-0.123; 0.036	-1.068	0.285
France	0.277	-0.066; 0.620	1.585	0.113	0.274	-0.065; 0.613	1.583	0.113
Germany	-0.088	-0.396; 0.219	-0.563	0.573	-0.096	-0.399; 0.208	-0.617	0.537
Italy	-0.232	-0.532; 0.068	-1.518	0.129	-0.236	-0.532; 0.060	-1.561	0.118
Netherlands	-0.155	-0.549; 0.240	-0.768	0.442	-0.161	-0.555; 0.234	-0.798	0.425
Poland	-0.170	-0.490; 0.151	-1.038	0.299	-0.195	-0.511; 0.122	-1.203	0.229
Turkey	-0.270	-0.588; 0.048	-1.662	0.097	-0.278	-0.594; 0.038	-1.727	0.084
United Kingdom	0.007	-0.307; 0.321	0.043	0.966	0.017	-0.293; 0.328	0.110	0.913

Supplementary Table 6. (continued)

	Salience + Age				Salience (WS/BS) + Age			
Predictors	b	95%-CI	*t*	*p*	b	95%-CI	*t*	*p*
(Intercept)	0.207	-0.117; 0.531	1.251	0.211	0.226	-0.095; 0.547	1.379	0.168
Tweets	-0.064	-0.091; -0.037	-4.705	<0.001				
Tweets (WS)					-0.068	-0.096; -0.040	-4.692	<0.001
Tweets (BS)					-0.025	-0.122; 0.072	-0.501	0.617
SDV	0.050	0.008; 0.093	2.331	0.020	0.056	0.011; 0.100	2.433	0.015
Tweets * SDV	-0.011	-0.037; 0.016	-0.778	0.436				
Tweets (WS) * SDV					-0.009	-0.038; 0.019	-0.635	0.525
Tweets (BS) * SDV					-0.015	-0.108; 0.079	-0.309	0.757
France	0.308	-0.068; 0.683	1.606	0.108	0.294	-0.079; 0.666	1.546	0.122
Germany	-0.013	-0.350; 0.325	-0.074	0.941	-0.033	-0.367; 0.301	-0.196	0.845
Italy	-0.344	-0.673; -0.014	-2.045	0.041	-0.359	-0.685; -0.033	-2.159	0.031
Netherlands	-0.148	-0.582; 0.286	-0.668	0.504	-0.178	-0.612; 0.257	-0.802	0.423
Poland	-0.255	-0.607; 0.096	-1.422	0.155	-0.287	-0.637; 0.062	-1.613	0.107
Turkey	-0.254	-0.603; 0.095	-1.424	0.154	-0.283	-0.630; 0.065	-1.596	0.111
United Kingdom	-0.035	-0.382; 0.312	-0.198	0.843	-0.034	-0.379; 0.311	-0.193	0.847

Supplementary Table 6. (continued)

	Salience + Gender				Salience (WS/BS) + Gender			
Predictors	b	95%-CI	*t*	*p*	b	95%-CI	*t*	*p*
(Intercept)	0.198	-0.133; 0.530	1.173	0.241	0.217	-0.111; 0.546	1.296	0.195
Tweets	-0.064	-0.092; -0.035	-4.315	<0.001				
Tweets (WS)					-0.064	-0.095; -0.034	-4.109	<0.001
Tweets (BS)					-0.053	-0.156; 0.050	-1.008	0.313
SDV	0.174	0.076; 0.271	3.489	<0.001	0.177	0.078; 0.276	3.496	<0.001
Tweets * SDV	-0.026	-0.094; 0.043	-0.741	0.459				
Tweets (WS) * SDV					-0.031	-0.104; 0.041	-0.849	0.396
Tweets (BS) * SDV					0.030	-0.210; 0.269	0.243	0.808
France	0.289	-0.092; 0.670	1.484	0.138	0.270	-0.108; 0.649	1.400	0.162
Germany	0.010	-0.334; 0.354	0.058	0.953	-0.010	-0.351; 0.331	-0.059	0.953
Italy	-0.388	-0.723; -0.053	-2.271	0.023	-0.407	-0.739; -0.074	-2.399	0.016
Netherlands	-0.142	-0.584; 0.301	-0.626	0.531	-0.167	-0.611; 0.277	-0.737	0.461
Poland	-0.252	-0.610; 0.105	-1.383	0.167	-0.278	-0.634; 0.078	-1.533	0.125
Turkey	-0.271	-0.627; 0.084	-1.496	0.135	-0.297	-0.651; 0.056	-1.648	0.099
United Kingdom	-0.071	-0.422; 0.280	-0.397	0.691	-0.084	-0.433; 0.264	-0.474	0.636

Supplementary Table 6. (continued)

	Salience + Political attitude				Salience (WS/BS) + Political attitude			
Predictors	b	95%-CI	*t*	*p*	b	95%-CI	*t*	*p*
(Intercept)	0.236	-0.087; 0.560	1.431	0.153	0.261	-0.061; 0.582	1.590	0.112
Tweets	-0.069	-0.095; -0.043	-5.120	<0.001				
Tweets (WS)					-0.072	-0.100; -0.044	-5.043	<0.001
Tweets (BS)					-0.035	-0.130; 0.060	-0.716	0.474
SDV	0.043	0.005; 0.081	2.203	0.028	0.048	0.008; 0.087	2.374	0.018
Tweets * SDV	-0.012	-0.040; 0.015	-0.887	0.375				
Tweets (WS) * SDV					-0.015	-0.045; 0.014	-1.035	0.301
Tweets (BS) * SDV					0.022	-0.073; 0.117	0.445	0.657
France	0.278	-0.097; 0.653	1.452	0.147	0.258	-0.115; 0.631	1.358	0.175
Germany	-0.007	-0.344; 0.330	-0.042	0.966	-0.029	-0.363; 0.305	-0.169	0.865
Italy	-0.390	-0.718; -0.062	-2.332	0.020	-0.413	-0.738; -0.087	-2.486	0.013
Netherlands	-0.144	-0.578; 0.289	-0.652	0.514	-0.173	-0.607; 0.262	-0.779	0.436
Poland	-0.257	-0.608; 0.094	-1.433	0.152	-0.289	-0.638; 0.061	-1.620	0.105
Turkey	-0.264	-0.613; 0.085	-1.485	0.138	-0.293	-0.640; 0.055	-1.652	0.099
United Kingdom	-0.095	-0.440; 0.249	-0.542	0.588	-0.106	-0.447; 0.235	-0.608	0.543

Supplementary Table 6. (continued)

	Salience + Social status				Salience (WS/BS) + Social status			
Predictors	b	95%-CI	*t*	*p*	b	95%-CI	*t*	*p*
(Intercept)	0.239	-0.081; 0.558	1.462	0.144	0.265	-0.052; 0.582	1.636	0.102
Tweets	-0.067	-0.093; -0.041	-5.006	<0.001				
Tweets (WS)					-0.070	-0.098; -0.042	-4.921	<0.001
Tweets (BS)					-0.037	-0.131; 0.056	-0.780	0.435
SDV	0.123	0.084; 0.162	6.235	<0.001	0.127	0.087; 0.167	6.243	<0.001
Tweets * SDV	0.004	-0.023; 0.031	0.293	0.769				
Tweets (WS) * SDV					0.001	-0.028; 0.030	0.071	0.944
Tweets (BS) * SDV					0.037	-0.057; 0.131	0.774	0.439
France	0.265	-0.106; 0.636	1.399	0.162	0.241	-0.127; 0.610	1.284	0.199
Germany	-0.058	-0.392; 0.275	-0.343	0.732	-0.081	-0.411; 0.249	-0.482	0.630
Italy	-0.367	-0.691; -0.043	-2.218	0.027	-0.391	-0.713; -0.070	-2.385	0.017
Netherlands	-0.214	-0.642; 0.215	-0.976	0.329	-0.253	-0.685; 0.178	-1.150	0.250
Poland	-0.265	-0.612; 0.082	-1.497	0.134	-0.299	-0.643; 0.046	-1.698	0.089
Turkey	-0.275	-0.620; 0.069	-1.566	0.117	-0.309	-0.652; 0.034	-1.766	0.077
United Kingdom	-0.096	-0.436; 0.244	-0.553	0.580	-0.111	-0.449; 0.227	-0.644	0.520

Notes. N = 1,341. Displays fixed-effect coefficients of multilevel models. All statistical tests were two-sided. The outcome of all models was well-being. Degrees of freedom were >10,000 for all statistical tests. WS = within-subjects component; BS = between-subjects component; b = unstandardized regression weight; 95%-CI = 95% confidence interval around the estimate; SDV = sociodemographic variable as indicated in the column head.

Supplementary Table 7. Coefficients and model fits of models representing different well-being trajectories excluding one country.

	Without France (N = 1,280)				Without Germany (N = 1,122)			
Predictors	b	95%-CI	*t*	*p*	b	95%-CI	*t*	*p*
(Intercept)	-0.100	-0.167; -0.033	-2.920	0.004	-0.122	-0.195; -0.049	-3.272	0.001
Level	-0.200	-0.279; -0.122	-5.021	<0.001	-0.190	-0.282; -0.099	-4.072	<0.001
Pre-event	-0.001	-0.104; 0.101	-0.027	0.978	0.005	-0.124; 0.133	0.072	0.943
Post-event	0.061	-0.035; 0.158	1.242	0.214	0.082	-0.023; 0.187	1.532	0.126

	Without Italy (N = 682)				Without the Netherlands (N = 1,319)			
Predictors	b	95%-CI	*t*	*p*	b	95%-CI	*t*	*p*
(Intercept)	-0.077	-0.170; 0.015	-1.642	0.101	-0.088	-0.155; -0.020	-2.557	0.011
Level	-0.249	-0.336; -0.162	-5.610	<0.001	-0.191	-0.270; -0.112	-4.729	<0.001
Pre-event	0.044	-0.060; 0.147	0.830	0.407	0.001	-0.103; 0.104	0.012	0.991
Post-event	0.424	0.282; 0.566	5.838	<0.001	0.085	-0.010; 0.180	1.764	0.078

	Without Poland (N = 1,238)				Without Turkey (N = 1,229)			
Predictors	b	95%-CI	*t*	*p*	b	95%-CI	*t*	*p*
(Intercept)	-0.060	-0.130; 0.010	-1.668	0.095	-0.086	-0.158; -0.014	-2.337	0.019
Level	-0.184	-0.266; -0.101	-4.345	<0.001	-0.274	-0.366; -0.181	-5.810	<0.001
Pre-event	0.034	-0.074; 0.142	0.614	0.539	0.055	-0.051; 0.162	1.019	0.308
Post-event	0.051	-0.047; 0.149	1.015	0.310	0.067	-0.032; 0.165	1.328	0.184

	Without the United Kingdom (N = 1,196)			
Predictors	b	95%-CI	*t*	*p*
(Intercept)	-0.109	-0.178; -0.041	-3.130	0.002
Level	-0.150	-0.234; -0.067	-3.523	<0.001
Pre-event	-0.123	-0.256; 0.011	-1.803	0.071
Post-event	0.096	-0.002; 0.194	1.917	0.055

Notes. Displays fixed-effect coefficients of multilevel models. All statistical tests were two-sided. The outcome of all models was well-being. The degrees of freedom were >9,000 for all statistical tests. b = unstandardized regression weight; 95%-CI = 95% confidence interval around the estimate.

Supplementary Table 8. Coefficients of Model 2d including additional level-2 predictors excluding one country.

	Without France (N = 1,280)				Without Germany (N = 1,122)			
Predictors	b	95%-CI	*t*	*p*	b	95%-CI	*t*	*p*
(Intercept)	-0.097	-0.163; -0.032	-2.925	0.003	-0.101	-0.172; -0.030	-2.787	0.005
Level	-0.182	-0.261; -0.103	-4.501	<0.001	-0.176	-0.268; -0.084	-3.762	<0.001
Pre-event	0.035	-0.068; 0.137	0.668	0.504	0.006	-0.122; 0.133	0.085	0.932
Post-event	0.099	0.005; 0.193	2.065	0.039	0.124	0.022; 0.226	2.386	0.017
Stability	0.242	0.176; 0.308	7.174	<0.001	0.248	0.175; 0.320	6.718	<0.001
Level * Stability	-0.027	-0.103; 0.048	-0.704	0.481	-0.006	-0.092; 0.081	-0.127	0.899
Pre-event * Stability	-0.045	-0.142; 0.051	-0.919	0.358	-0.055	-0.176; 0.067	-0.885	0.376
Post-event * Stability	0.154	0.057; 0.251	3.104	0.002	0.168	0.063; 0.274	3.135	0.002

	Without Italy (N = 682)				Without the Netherlands (N = 1,319)			
Predictors	b	95%-CI	*t*	*p*	b	95%-CI	*t*	*p*
(Intercept)	-0.145	-0.235; -0.055	-3.149	0.002	-0.086	-0.151; -0.020	-2.564	0.010
Level	-0.257	-0.345; -0.168	-5.678	<0.001	-0.172	-0.252; -0.093	-4.237	<0.001
Pre-event	0.055	-0.049; 0.158	1.038	0.300	0.038	-0.066; 0.141	0.716	0.474
Post-event	0.431	0.288; 0.575	5.889	<0.001	0.117	0.025; 0.209	2.490	0.013
Stability	0.301	0.217; 0.385	7.026	<0.001	0.245	0.179; 0.311	7.257	<0.001
Level * Stability	0.019	-0.063; 0.102	0.463	0.643	-0.019	-0.095; 0.056	-0.505	0.614
Pre-event * Stability	-0.031	-0.129; 0.066	-0.630	0.528	-0.052	-0.149; 0.046	-1.036	0.300
Post-event * Stability	-0.063	-0.201; 0.075	-0.890	0.373	0.165	0.070; 0.260	3.414	0.001

	Without Poland (N = 1,238)				Without Turkey (N = 1,229)			
Predictors	b	95%-CI	*t*	*p*	b	95%-CI	*t*	*p*
(Intercept)	-0.061	-0.130; 0.007	-1.758	0.079	-0.074	-0.145; -0.003	-2.041	0.041
Level	-0.158	-0.242; -0.073	-3.642	<0.001	-0.241	-0.333; -0.148	-5.095	<0.001
Pre-event	0.061	-0.047; 0.168	1.106	0.269	0.093	-0.013; 0.199	1.727	0.084
Post-event	0.083	-0.012; 0.179	1.717	0.086	0.092	-0.004; 0.188	1.871	0.061
Stability	0.249	0.179; 0.319	6.975	<0.001	0.228	0.155; 0.302	6.096	<0.001
Level * Stability	-0.038	-0.120; 0.044	-0.912	0.362	-0.064	-0.154; 0.026	-1.388	0.165
Pre-event * Stability	-0.026	-0.130; 0.078	-0.487	0.626	-0.024	-0.124; 0.077	-0.463	0.643
Post-event * Stability	0.155	0.056; 0.254	3.078	0.002	0.183	0.082; 0.283	3.551	<0.001

Supplementary Table 8. (continued)

	**Without the United Kingdom** (N = 1,196)			
Predictors	b	95%-CI	t	p
(Intercept)	-0.110	-0.176; -0.043	-3.229	0.001
Level	-0.136	-0.221; -0.050	-3.116	0.002
Pre-event	-0.029	-0.168; 0.110	-0.408	0.683
Post-event	0.133	0.038; 0.228	2.736	0.006
Stability	0.237	0.169; 0.305	6.806	<0.001
Level * Stability	-0.026	-0.108; 0.055	-0.635	0.525
Pre-event * Stability	-0.066	-0.193; 0.060	-1.029	0.303
Post-event * Stability	0.194	0.094; 0.293	3.797	<0.001

Notes. Displays fixed-effect coefficients of multilevel models. All statistical tests were two-sided. The outcome of all models was well-being. The degrees of freedom were >9,000 for all statistical tests. b = unstandardized regression weight; 95%-CI = 95% confidence interval around the estimate.

1.3. Results of supplementary analyses including global data

Supplementary Table 9. Coefficients of Model 2d including additional level-2 predictors in global data.

	**Model 2d**				**Model 2d + Stability**			
Predictors	b	95%-CI	t	p	b	95%-CI	t	p
(Intercept)	0.117	0.032; 0.202	2.684	0.007	-0.079	-0.160; 0.002	-1.906	0.057
Level	-0.149	-0.210; -0.087	-4.740	<0.001	-0.136	-0.199; -0.073	-4.248	<0.001
Pre-event	-0.051	-0.140; 0.038	-1.130	0.259	-0.034	-0.122; 0.054	-0.758	0.449
Post-event	0.063	-0.017; 0.144	1.543	0.123	0.091	0.013; 0.169	2.297	0.022
Stability					0.314	0.259; 0.369	11.149	<0.001
Level * Stability					-0.036	-0.096; 0.024	-1.162	0.245
Pre-event * Stability					0.015	-0.072; 0.103	0.343	0.731
Post-event * Stability					0.074	-0.003; 0.152	1.876	0.061
European	-0.245	-0.329; -0.162	-5.744	<0.001	0.002	-0.076; 0.080	0.054	0.957

Supplementary Table 9. (continued)

	Model 2d + Age				Model 2d + Gender			
Predictors	b	95%-CI	*t*	*p*	b	95%-CI	*t*	*p*
(Intercept)	0.075	-0.014; 0.164	1.648	0.099	0.090	-0.001; 0.182	1.931	0.053
Level	-0.148	-0.211; -0.086	-4.648	<0.001	-0.148	-0.217; -0.078	-4.172	<0.001
Pre-event	-0.032	-0.117; 0.054	-0.724	0.469	-0.057	-0.156; 0.042	-1.130	0.258
Post-event	0.074	-0.006; 0.155	1.806	0.071	0.060	-0.030; 0.151	1.311	0.190
SDV	0.029	-0.032; 0.089	0.930	0.352	0.125	-0.017; 0.268	1.720	0.085
Level * SDV	0.019	-0.041; 0.080	0.623	0.533	-0.004	-0.155; 0.147	-0.049	0.961
Pre-event * SDV	-0.161	-0.245; -0.078	-3.791	<0.001	-0.052	-0.285; 0.180	-0.440	0.660
Post-event * SDV	0.043	-0.039; 0.126	1.036	0.300	0.017	-0.186; 0.220	0.166	0.868
European	-0.196	-0.286; -0.106	-4.255	<0.001	-0.244	-0.328; -0.160	-5.675	<0.001

	Model 2d + Political attitude				Model 2d + Social status			
Predictors	b	95%-CI	*t*	*p*	b	95%-CI	*t*	*p*
(Intercept)	0.107	0.022; 0.192	2.455	0.014	0.111	0.026; 0.196	2.560	0.010
Level	-0.152	-0.213; -0.091	-4.873	<0.001	-0.151	-0.212; -0.089	-4.788	<0.001
Pre-event	-0.052	-0.141; 0.037	-1.145	0.252	-0.024	-0.114; 0.067	-0.517	0.605
Post-event	0.064	-0.016; 0.145	1.561	0.119	0.084	0.004; 0.165	2.059	0.039
SDV	0.009	-0.048; 0.066	0.306	0.760	0.073	0.016; 0.130	2.526	0.012
Level * SDV	-0.066	-0.126; -0.006	-2.172	0.030	0.010	-0.051; 0.071	0.325	0.746
Pre-event * SDV	-0.023	-0.117; 0.070	-0.491	0.623	-0.066	-0.162; 0.030	-1.344	0.179
Post-event * SDV	0.041	-0.040; 0.122	0.985	0.325	0.099	0.019; 0.179	2.426	0.015
European	-0.234	-0.318; -0.150	-5.485	<0.001	-0.239	-0.321; -0.156	-5.649	<0.001

Notes. N = 1,735. Displays fixed-effect coefficients of multilevel models. All statistical tests were two-sided. The outcome of all models was well-being. Degrees of freedom were >10,000 for all statistical tests. b = unstandardized regression weight; 95%-CI = 95% confidence interval around the estimate; SDV = sociodemographic variable as indicated in the column head.

Supplementary Table 10. Predicting well-being by the salience of the war and additional level-2 predictors in global data.

	Salience				Salience (WS/BS)			
Predictors	b	95%-CI	*t*	*p*	b	95%-CI	*t*	*p*
(Intercept)	0.192	0.117; 0.266	5.056	<0.001	0.189	0.112; 0.265	4.854	<0.001
Tweets	-0.050	-0.069; -0.030	-5.007	<0.001				
Tweets (WS)					-0.050	-0.071; -0.030	-4.946	<0.001
Tweets (BS)					-0.016	-0.086; 0.054	-0.448	0.654
European	-0.249	-0.333; -0.165	-5.799	<0.001	-0.243	-0.330; -0.156	-5.468	<0.001

	Salience + Stability				Salience (WS/BS) + Stability			
Predictors	b	95%-CI	*t*	*p*	b	95%-CI	*t*	*p*
(Intercept)	-0.013	-0.081; 0.055	-0.372	0.710	-0.002	-0.073; 0.068	-0.069	0.945
Tweets	-0.057	-0.076; -0.037	-5.614	<0.001				
Tweets (WS)					-0.056	-0.077; -0.035	-5.301	<0.001
Tweets (BS)					-0.055	-0.119; 0.008	-1.700	0.089
Stability	0.363	0.331; 0.395	22.002	<0.001	0.364	0.332; 0.397	22.089	<0.001
Tweets * Stability	0.014	-0.004; 0.032	1.496	0.135				
Tweets (WS) * Stability					0.016	-0.003; 0.035	1.690	0.091
Tweets (BS) * Stability					-0.007	-0.063; 0.049	-0.249	0.803
European	0.014	-0.064; 0.093	0.360	0.719	0.004	-0.077; 0.085	0.097	0.923

	Salience + Age				Salience (WS/BS) + Age			
Predictors	b	95%-CI	*t*	*p*	b	95%-CI	*t*	*p*
(Intercept)	0.131	0.053; 0.209	3.281	0.001	0.120	0.039; 0.200	2.902	0.004
Tweets	-0.050	-0.071; -0.030	-4.905	<0.001				
Tweets (WS)					-0.051	-0.072; -0.030	-4.807	<0.001
Tweets (BS)					-0.025	-0.097; 0.047	-0.689	0.491
SDV	0.090	0.053; 0.128	4.702	<0.001	0.095	0.057; 0.132	4.926	<0.001
Tweets * SDV	0.003	-0.016; 0.022	0.342	0.732				
Tweets (WS) * SDV					0.002	-0.018; 0.021	0.179	0.858
Tweets (BS) * SDV					0.036	-0.028; 0.100	1.098	0.272
European	-0.168	-0.258; -0.078	-3.656	<0.001	-0.154	-0.247; -0.060	-3.223	0.001

Supplementary Table 10. (continued)

	Salience + Gender				Salience (WS/BS) + Gender			
Predictors	b	95%-CI	*t*	*p*	b	95%-CI	*t*	*p*
(Intercept)	0.162	0.085; 0.240	4.104	<0.001	0.163	0.083; 0.242	4.014	<0.001
Tweets	-0.050	-0.072; -0.028	-4.468	<0.001				
Tweets (WS)					-0.051	-0.073; -0.028	-4.364	<0.001
Tweets (BS)					-0.022	-0.100; 0.057	-0.541	0.588
SDV	0.143	0.056; 0.230	3.221	0.001	0.145	0.058; 0.232	3.256	0.001
Tweets * SDV	-0.001	-0.048; 0.046	-0.045	0.964				
Tweets (WS) * SDV					0.001	-0.048; 0.049	0.029	0.977
Tweets (BS) * SDV					-0.028	-0.191; 0.135	-0.337	0.736
European	-0.246	-0.331; -0.161	-5.704	<0.001	-0.244	-0.332; -0.157	-5.464	<0.001

	Salience + Political attitude				Salience (WS/BS) + Political attitude			
Predictors	b	95%-CI	*t*	*p*	b	95%-CI	*t*	*p*
(Intercept)	0.184	0.110; 0.258	4.852	<0.001	0.180	0.104; 0.257	4.639	<0.001
Tweets	-0.051	-0.070; -0.031	-5.131	<0.001				
Tweets (WS)					-0.052	-0.072; -0.032	-5.087	<0.001
Tweets (BS)					-0.014	-0.083; 0.056	-0.380	0.704
SDV	0.049	0.014; 0.084	2.751	0.006	0.052	0.016; 0.087	2.877	0.004
Tweets * SDV	-0.018	-0.037; 0.000	-1.931	0.053				
Tweets (WS) * SDV					-0.018	-0.037; 0.001	-1.838	0.066
Tweets (BS) * SDV					-0.019	-0.082; 0.044	-0.592	0.554
European	-0.239	-0.323; -0.155	-5.561	<0.001	-0.232	-0.320; -0.145	-5.214	<0.001

Supplementary Table 10. (continued)

	Salience + Social status				Salience (WS/BS) + Social status			
Predictors	b	95%-CI	*t*	*p*	b	95%-CI	*t*	*p*
(Intercept)	0.184	0.111; 0.257	4.932	<0.001	0.179	0.104; 0.254	4.690	<0.001
Tweets	-0.049	-0.068; -0.029	-4.902	<0.001				
Tweets (WS)					-0.050	-0.070; -0.030	-4.898	<0.001
Tweets (BS)					-0.009	-0.078; 0.060	-0.254	0.800
SDV	0.131	0.096; 0.165	7.422	<0.001	0.132	0.097; 0.166	7.466	<0.001
Tweets * SDV	-0.009	-0.028; 0.009	-0.997	0.319				
Tweets (WS) * SDV					-0.010	-0.029; 0.010	-0.972	0.331
Tweets (BS) * SDV					-0.002	-0.065; 0.061	-0.062	0.951
European	-0.236	-0.319; -0.153	-5.585	<0.001	-0.228	-0.314; -0.142	-5.214	<0.001

Notes. $N = 1{,}735$. Displays fixed-effect coefficients of multilevel models. All statistical tests were two-sided. The outcome of all models was well-being. Degrees of freedom were >10,000 for all statistical tests. WS = within-subjects component; BS = between-subjects component; b = unstandardized regression weight; 95%-CI = 95% confidence interval around the estimate; SDV = sociodemographic variable as indicated in the column head.

1.4. Results of supplementary analyses with different time frames

Supplementary Table 11. Coefficients of Model 2d including additional level-2 predictors with shorter time frame.

	Model 2d				Model 2d + Stability			
Predictors	b	95%-CI	*t*	*p*	b	95%-CI	*t*	*p*
(Intercept)	-0.215	-0.343; -0.086	-3.281	0.001	-0.229	-0.352; -0.106	-3.658	<0.001
Level	-0.356	-0.504; -0.207	-4.699	<0.001	-0.354	-0.504; -0.205	-4.652	<0.001
Pre-event	0.149	-0.639; 0.937	0.372	0.710	0.062	-0.718; 0.842	0.155	0.877
Post-event	1.120	0.253; 1.987	2.533	0.011	1.165	0.296; 2.034	2.629	0.009
Stability					0.305	0.181; 0.429	4.830	<0.001
Level * Stability					-0.045	-0.193; 0.104	-0.587	0.557
Pre-event * Stability					1.006	0.228; 1.785	2.534	0.011
Post-event * Stability					-0.289	-1.186; 0.608	-0.632	0.528

Supplementary Table 11. (continued)

	Model 2d + Age				Model 2d + Gender			
Predictors	b	95%-CI	*t*	*p*	b	95%-CI	*t*	*p*
(Intercept)	-0.217	-0.345; -0.089	-3.329	0.001	-0.197	-0.338; -0.056	-2.732	0.006
Level	-0.359	-0.508; -0.211	-4.743	<0.001	-0.327	-0.500; -0.155	-3.728	<0.001
Pre-event	0.148	-0.641; 0.936	0.368	0.713	0.082	-0.818; 0.981	0.178	0.859
Post-event	1.136	0.270; 2.003	2.572	0.010	1.001	0.045; 1.957	2.054	0.040
SDV	0.064	-0.062; 0.189	0.996	0.319	-0.105	-0.442; 0.233	-0.607	0.544
Level * SDV	0.081	-0.064; 0.226	1.100	0.272	-0.145	-0.549; 0.259	-0.705	0.481
Pre-event * SDV	-0.324	-1.117; 0.469	-0.801	0.423	0.371	-1.707; 2.450	0.350	0.726
Post-event * SDV	-0.308	-1.163; 0.548	-0.705	0.481	0.802	-1.500; 3.104	0.683	0.495

	Model 2d + Political attitude				Model 2d + Social status			
Predictors	b	95%-CI	*t*	*p*	b	95%-CI	*t*	*p*
(Intercept)	-0.217	-0.346; -0.088	-3.302	0.001	-0.212	-0.338; -0.086	-3.302	0.001
Level	-0.359	-0.514; -0.205	-4.574	<0.001	-0.353	-0.510; -0.197	-4.421	<0.001
Pre-event	0.165	-0.623; 0.954	0.411	0.681	0.162	-0.647; 0.970	0.392	0.695
Post-event	1.139	0.264; 2.013	2.554	0.011	1.117	0.246; 1.988	2.515	0.012
SDV	-0.025	-0.159; 0.110	-0.357	0.721	0.179	0.050; 0.309	2.713	0.007
Level * SDV	-0.063	-0.224; 0.098	-0.769	0.442	0.041	-0.118; 0.200	0.508	0.612
Pre-event * SDV	-0.003	-0.821; 0.816	-0.006	0.995	0.484	-0.327; 1.296	1.170	0.242
Post-event * SDV	0.274	-0.633; 1.180	0.592	0.554	-0.309	-1.192; 0.575	-0.685	0.493

Notes. N = 341. Displays fixed-effect coefficients of multilevel models. All statistical tests were two-sided. The outcome of all models was well-being. Degrees of freedom were >2,000 for all statistical tests. b = unstandardized regression weight; 95%-CI = 95% confidence interval around the estimate; SDV = sociodemographic variable as indicated in the column head.

Supplementary Table 12. Predicting well-being by the salience of the war and additional level-2 predictors with shorter time frame.

	Salience				Salience (WS/BS)			
Predictors	b	95%-CI	*t*	*p*	b	95%-CI	*t*	*p*
(Intercept)	-0.006	-0.087; 0.076	-0.134	0.893	-0.005	-0.089; 0.080	-0.109	0.913
Tweets	-0.134	-0.182; -0.086	-5.473	<0.001				
Tweets (WS)					-0.133	-0.184; -0.083	-5.159	<0.001
Tweets (BS)					-0.143	-0.286; 0.001	-1.950	0.051

	Salience + Stability				Salience (WS/BS) + Stability			
Predictors	b	95%-CI	*t*	*p*	b	95%-CI	*t*	*p*
(Intercept)	-0.012	-0.089; 0.064	-0.319	0.749	-0.010	-0.090; 0.069	-0.260	0.795
Tweets	-0.137	-0.185; -0.089	-5.625	<0.001	-0.135	-0.185; -0.084	-5.212	<0.001
Tweets (WS)					-0.144	-0.279; -0.008	-2.080	0.038
Tweets (BS)					0.263	0.183; 0.344	6.420	<0.001
Stability	0.253	0.176; 0.329	6.495	<0.001				
Tweets * Stability	0.021	-0.028; 0.069	0.841	0.400				
Tweets (WS) * Stability					0.015	-0.036; 0.066	0.566	0.571
Tweets (BS) * Stability					0.080	-0.061; 0.222	1.114	0.265

	Salience + Age				Salience (WS/BS) + Age			
Predictors	b	95%-CI	*t*	*p*	b	95%-CI	*t*	*p*
(Intercept)	-0.006	-0.087; 0.075	-0.144	0.886	-0.005	-0.089; 0.079	-0.110	0.912
Tweets	-0.135	-0.183; -0.087	-5.507	<0.001				
Tweets (WS)					-0.134	-0.185; -0.083	-5.166	<0.001
Tweets (BS)					-0.125	-0.272; 0.023	-1.660	0.097
SDV	0.027	-0.055; 0.109	0.646	0.518	0.038	-0.046; 0.123	0.892	0.372
Tweets * SDV	0.013	-0.034; 0.059	0.546	0.585				
Tweets (WS) * SDV					0.006	-0.042; 0.055	0.258	0.796
Tweets (BS) * SDV					0.103	-0.066; 0.273	1.194	0.233

Supplementary Table 12. (continued)

	Salience + Gender				Salience (WS/BS) + Gender			
Predictors	b	95%-CI	*t*	*p*	b	95%-CI	*t*	*p*
(Intercept)	-0.003	-0.093; 0.086	-0.069	0.945	-0.001	-0.094; 0.091	-0.025	0.980
Tweets	-0.128	-0.182; -0.075	-4.743	<0.001				
Tweets (WS)					-0.128	-0.184; -0.072	-4.468	<0.001
Tweets (BS)					-0.136	-0.292; 0.020	-1.704	0.089
SDV	-0.013	-0.226; 0.201	-0.117	0.907	-0.007	-0.232; 0.218	-0.064	0.949
Tweets * SDV	-0.025	-0.150; 0.100	-0.389	0.697				
Tweets (WS) * SDV					-0.031	-0.161; 0.100	-0.460	0.646
Tweets (BS) * SDV					0.036	-0.362; 0.434	0.178	0.859

	Salience + Political attitude				Salience (WS/BS) + Political attitude			
Predictors	b	95%-CI	*t*	*p*	b	95%-CI	*t*	*p*
(Intercept)	-0.006	-0.088; 0.075	-0.148	0.883	0.003	-0.082; 0.088	0.069	0.945
Tweets	-0.135	-0.183; -0.087	-5.513	<0.001				
Tweets (WS)					-0.135	-0.186; -0.085	-5.245	<0.001
Tweets (BS)					-0.135	-0.278; 0.009	-1.843	0.065
SDV	0.014	-0.070; 0.098	0.327	0.743	0.045	-0.045; 0.135	0.973	0.331
Tweets * SDV	-0.022	-0.072; 0.028	-0.868	0.385				
Tweets (WS) * SDV					-0.037	-0.089; 0.015	-1.395	0.163
Tweets (BS) * SDV					0.116	-0.034; 0.265	1.515	0.130

	Salience + Social status				Salience (WS/BS) + Social status			
Predictors	b	95%-CI	*t*	*p*	b	95%-CI	*t*	*p*
(Intercept)	-0.004	-0.085; 0.076	-0.107	0.915	-0.001	-0.085; 0.082	-0.035	0.972
Tweets	-0.133	-0.181; -0.085	-5.470	<0.001				
Tweets (WS)					-0.133	-0.184; -0.083	-5.195	<0.001
Tweets (BS)					-0.132	-0.274; 0.010	-1.824	0.068
SDV	0.117	0.035; 0.198	2.812	0.005	0.117	0.031; 0.202	2.671	0.008
Tweets * SDV	0.041	-0.007; 0.090	1.661	0.097				
Tweets (WS) * SDV					0.040	-0.011; 0.091	1.527	0.127
Tweets (BS) * SDV					0.050	-0.101; 0.201	0.645	0.519

Notes. N = 341. Displays fixed-effect coefficients of multilevel models. All statistical tests were two-sided. The outcome of all models was well-being. Degrees of freedom were >2,000 for all statistical tests. WS = within-subjects component; BS = between-subjects component;

b = unstandardized regression weight; 95%-CI = 95% confidence interval around the estimate; SDV = sociodemographic variable as indicated in the column head.

Supplementary Table 13. Coefficients of Model 2d including additional level-2 predictors with longer time frame.

	Model 2d				Model 2d + Stability			
Predictors	b	95%-CI	*t*	*p*	b	95%-CI	*t*	*p*
(Intercept)	-0.086	-0.139; -0.032	-3.136	0.002	-0.063	-0.115; -0.012	-2.413	0.016
Level	-0.141	-0.211; -0.072	-3.986	<0.001	-0.112	-0.181; -0.043	-3.178	0.001
Pre-event	-0.082	-0.153; -0.012	-2.309	0.021	-0.061	-0.129; 0.007	-1.760	0.078
Post-event	0.084	0.035; 0.133	3.388	0.001	0.073	0.026; 0.120	3.040	0.002
Stability					0.291	0.239; 0.344	10.810	<0.001
Level * Stability					0.003	-0.064; 0.070	0.087	0.931
Pre-event * Stability					-0.020	-0.086; 0.045	-0.600	0.548
Post-event * Stability					0.071	0.023; 0.120	2.892	0.004

	Model 2d + Age				Model 2d + Gender			
Predictors	b	95%-CI	*t*	*p*	b	95%-CI	*t*	*p*
(Intercept)	-0.082	-0.137; -0.028	-2.991	0.003	-0.125	-0.184; -0.065	-4.091	<0.001
Level	-0.134	-0.204; -0.064	-3.732	<0.001	-0.156	-0.233; -0.079	-3.982	<0.001
Pre-event	-0.049	-0.120; 0.022	-1.357	0.175	-0.097	-0.173; -0.021	-2.515	0.012
Post-event	0.089	0.040; 0.138	3.544	<0.001	0.096	0.042; 0.150	3.465	0.001
SDV	0.044	-0.014; 0.102	1.481	0.139	0.190	0.053; 0.328	2.712	0.007
Level * SDV	-0.021	-0.090; 0.049	-0.579	0.563	0.065	-0.118; 0.248	0.696	0.486
Pre-event * SDV	-0.044	-0.097; 0.008	-1.655	0.098	0.055	-0.146; 0.255	0.533	0.594
Post-event * SDV	0.041	-0.011; 0.094	1.535	0.125	-0.041	-0.166; 0.084	-0.641	0.521

	Model 2d + Political attitude				Model 2d + Social status			
Predictors	b	95%-CI	*t*	*p*	b	95%-CI	*t*	*p*
(Intercept)	-0.087	-0.141; -0.034	-3.189	0.001	-0.075	-0.129; -0.022	-2.757	0.006
Level	-0.144	-0.214; -0.075	-4.071	<0.001	-0.140	-0.210; -0.069	-3.884	<0.001
Pre-event	-0.083	-0.153; -0.013	-2.327	0.020	-0.043	-0.110; 0.025	-1.239	0.215
Post-event	0.085	0.036; 0.133	3.414	0.001	0.085	0.036; 0.133	3.406	0.001
SDV	0.050	-0.005; 0.105	1.786	0.074	0.139	0.085; 0.192	5.080	<0.001
Level * SDV	-0.030	-0.103; 0.042	-0.821	0.411	0.055	-0.015; 0.125	1.533	0.125
Pre-event * SDV	0.014	-0.063; 0.091	0.362	0.718	-0.049	-0.115; 0.018	-1.441	0.150
Post-event * SDV	-0.011	-0.060; 0.039	-0.421	0.674	0.008	-0.041; 0.057	0.315	0.753

Notes. N = 1,915. Displays fixed-effect coefficients of multilevel models. All statistical tests were two-sided. The outcome of all models was well-being. Degrees of freedom were >10,000 for all

statistical tests. b = unstandardized regression weight; 95%-CI = 95% confidence interval around the estimate; SDV = sociodemographic variable as indicated in the column head.

Supplementary Table 14. Predicting well-being by the salience of the war and additional level-2 predictors with longer time frame.

	**Salience**				**Salience (WS/BS)**			
Predictors	b	95%-CI	*t*	*p*	b	95%-CI	*t*	*p*
(Intercept)	0.034	0.001; 0.067	2.002	0.045	0.037	0.004; 0.071	2.221	0.026
Tweets	-0.055	-0.076; -0.033	-4.864	<0.001				
Tweets (WS)					-0.032	-0.057; -0.007	-2.493	0.013
Tweets (BS)					-0.185	-0.255; -0.116	-5.230	<0.001

	**Salience + Stability**				**Salience (WS/BS) + Stability**			
Predictors	b	95%-CI	*t*	*p*	b	95%-CI	*t*	*p*
(Intercept)	0.033	0.004; 0.062	2.229	0.026	0.035	0.006; 0.064	2.381	0.017
Tweets	-0.053	-0.075; -0.032	-4.883	<0.001				
Tweets (WS)					-0.035	-0.060; -0.010	-2.743	0.006
Tweets (BS)					-0.149	-0.211; -0.088	-4.749	<0.001
Stability	0.352	0.323; 0.381	23.507	<0.001	0.352	0.323; 0.381	23.624	<0.001
Tweets * Stability	-0.021	-0.042; -0.000	-2.000	0.045				
Tweets (WS) * Stability					-0.022	-0.046; 0.002	-1.772	0.076
Tweets (BS) * Stability					-0.027	-0.088; 0.033	-0.880	0.379

	**Salience + Age**				**Salience (WS/BS) + Age**			
Predictors	b	95%-CI	*t*	*p*	b	95%-CI	*t*	*p*
(Intercept)	0.035	0.002; 0.068	2.083	0.037	0.040	0.007; 0.073	2.367	0.018
Tweets	-0.052	-0.074; -0.030	-4.638	<0.001				
Tweets (WS)					-0.032	-0.057; -0.007	-2.508	0.012
Tweets (BS)					-0.172	-0.242; -0.102	-4.847	<0.001
SDV	0.098	0.065; 0.131	5.835	<0.001	0.100	0.067; 0.134	5.870	<0.001
Tweets * SDV	-0.015	-0.037; 0.008	-1.300	0.194				
Tweets (WS) * SDV					-0.022	-0.047; 0.004	-1.637	0.102
Tweets (BS) * SDV					0.033	-0.037; 0.103	0.927	0.354

Supplementary Table 14. (continued)

	**Salience + Gender**				**Salience (WS/BS) + Gender**			
Predictors	b	95%-CI	*t*	*p*	b	95%-CI	*t*	*p*
(Intercept)	0.012	-0.025; 0.049	0.624	0.533	0.016	-0.021; 0.053	0.845	0.398
Tweets	-0.056	-0.080; -0.031	-4.489	<0.001				
Tweets (WS)					-0.029	-0.057; -0.002	-2.083	0.037
Tweets (BS)					-0.205	-0.281; -0.129	-5.262	<0.001
SDV	0.128	0.044; 0.213	2.978	0.003	0.127	0.042; 0.211	2.942	0.003
Tweets * SDV	-0.001	-0.059; 0.056	-0.040	0.968				
Tweets (WS) * SDV					-0.016	-0.081; 0.049	-0.493	0.622
Tweets (BS) * SDV					0.084	-0.099; 0.267	0.898	0.369

	**Salience + Political attitude**				**Salience (WS/BS) + Political attitude**			
Predictors	b	95%-CI	*t*	*p*	b	95%-CI	*t*	*p*
(Intercept)	0.034	0.001; 0.067	2.008	0.045	0.038	0.004; 0.071	2.225	0.026
Tweets	-0.054	-0.076; -0.032	-4.826	<0.001				
Tweets (WS)					-0.032	-0.057; -0.007	-2.474	0.013
Tweets (BS)					-0.183	-0.254; -0.113	-5.106	<0.001
SDV	0.040	0.007; 0.074	2.374	0.018				
Tweets * SDV	-0.002	-0.025; 0.021	-0.169	0.866	0.039	0.006; 0.073	2.311	0.021
Tweets (WS) * SDV					-0.001	-0.028; 0.025	-0.109	0.913
Tweets (BS) * SDV					-0.003	-0.079; 0.074	-0.068	0.946

	**Salience + Social status**				**Salience (WS/BS) + Social status**			
Predictors	b	95%-CI	*t*	*p*	b	95%-CI	*t*	*p*
(Intercept)	0.036	0.004; 0.069	2.179	0.029	0.040	0.007; 0.072	2.400	0.016
Tweets	-0.053	-0.075; -0.031	-4.782	<0.001				
Tweets (WS)					-0.033	-0.058; -0.008	-2.608	0.009
Tweets (BS)					-0.167	-0.236; -0.099	-4.770	<0.001
SDV	0.143	0.110; 0.176	8.586	<0.001	0.139	0.106; 0.172	8.353	<0.001
Tweets * SDV	0.001	-0.021; 0.023	0.075	0.940				
Tweets (WS) * SDV					-0.001	-0.027; 0.024	-0.109	0.913
Tweets (BS) * SDV					0.029	-0.042; 0.101	0.811	0.417

Notes. N = 1,915. Displays fixed-effect coefficients of multilevel models. All statistical tests were two-sided. The outcome of all models was well-being. Degrees of freedom were >10,000 for all statistical tests. WS = within-subjects component; BS = between-subjects component;

b = unstandardized regression weight; 95%-CI = 95% confidence interval around the estimate; SDV = sociodemographic variable as indicated in the column head.

1.5. Results of supplementary analyses including lagged effects of the salience

Supplementary Table 15. Predicting time-lagged well-being by the salience of the war and additional level-2 predictors.

	**Salience**				**Salience (WS/BS)**			
Predictors	b	95%-CI	*t*	*p*	b	95%-CI	*t*	*p*
(Intercept)	-0.004	-0.044; 0.036	-0.211	0.833	-0.009	-0.050; 0.031	-0.454	0.650
Tweets	-0.044	-0.069; -0.019	-3.397	0.001				
Tweets (WS)					-0.037	-0.064; -0.010	-2.671	0.008
Tweets (BS)					-0.110	-0.204; -0.015	-2.272	0.023

	**Salience + Stability**				**Salience (WS/BS) + Stability**			
Predictors	b	95%-CI	*t*	*p*	b	95%-CI	*t*	*p*
(Intercept)	-0.003	-0.039; 0.033	-0.156	0.876	-0.007	-0.043; 0.029	-0.383	0.702
Tweets	-0.044	-0.070; -0.019	-3.417	0.001				
Tweets (WS)					-0.038	-0.066; -0.011	-2.736	0.006
Tweets (BS)					-0.096	-0.182; -0.011	-2.201	0.028
Stability	0.335	0.299; 0.371	18.246	<0.001	0.333	0.296; 0.370	17.794	<0.001
Tweets * Stability	-0.010	-0.034; 0.014	-0.816	0.415				
Tweets (WS) * Stability					-0.007	-0.033; 0.019	-0.506	0.613
Tweets (BS) * Stability					-0.042	-0.125; 0.041	-0.988	0.323

	**Salience + Age**				**Salience (WS/BS) + Age**			
Predictors	b	95%-CI	*t*	*p*	b	95%-CI	*t*	*p*
(Intercept)	-0.001	-0.041; 0.039	-0.055	0.956	-0.005	-0.046; 0.035	-0.257	0.797
Tweets	-0.043	-0.069; -0.017	-3.237	0.001				
Tweets (WS)					-0.036	-0.064; -0.009	-2.582	0.010
Tweets (BS)					-0.102	-0.197; -0.006	-2.090	0.037
SDV	0.082	0.041; 0.123	3.894	<0.001	0.085	0.041; 0.129	3.761	<0.001
Tweets * SDV	-0.007	-0.033; 0.019	-0.517	0.605				
Tweets (WS) * SDV					-0.009	-0.037; 0.019	-0.635	0.525
Tweets (BS) * SDV					0.014	-0.081; 0.110	0.295	0.768

Supplementary Table 15. (continued)

	Salience + Gender				Salience (WS/BS) + Gender			
Predictors	b	95%-CI	*t*	*p*	b	95%-CI	*t*	*p*
(Intercept)	-0.029	-0.074; 0.015	-1.293	0.196	-0.036	-0.082; 0.009	-1.572	0.116
Tweets	-0.043	-0.071; -0.016	-3.052	0.002				
Tweets (WS)					-0.034	-0.064; -0.004	-2.222	0.026
Tweets (BS)					-0.133	-0.237; -0.030	-2.528	0.011
SDV	0.134	0.031; 0.237	2.558	0.011	0.143	0.038; 0.247	2.677	0.007
Tweets * SDV	-0.010	-0.077; 0.058	-0.288	0.774				
Tweets (WS) * SDV					-0.022	-0.094; 0.050	-0.603	0.547
Tweets (BS) * SDV					0.112	-0.147; 0.370	0.848	0.397

	Salience + Political attitude				Salience (WS/BS) + Political attitude			
Predictors	b	95%-CI	*t*	*p*	b	95%-CI	*t*	*p*
(Intercept)	-0.004	-0.044; 0.036	-0.202	0.840	-0.008	-0.049; 0.032	-0.400	0.689
Tweets	-0.044	-0.069; -0.018	-3.342	0.001				
Tweets (WS)					-0.037	-0.065; -0.010	-2.664	0.008
Tweets (BS)					-0.103	-0.199; -0.007	-2.095	0.036
SDV	0.037	-0.003; 0.077	1.837	0.066	0.038	-0.003; 0.080	1.825	0.068
Tweets * SDV	-0.002	-0.029; 0.025	-0.137	0.891				
Tweets (WS) * SDV					-0.003	-0.031; 0.026	-0.200	0.842
Tweets (BS) * SDV					0.015	-0.086; 0.116	0.291	0.771

	Salience + Social status				Salience (WS/BS) + Social status			
Predictors	b	95%-CI	*t*	*p*	b	95%-CI	*t*	*p*
(Intercept)	-0.001	-0.040; 0.038	-0.042	0.967	-0.005	-0.045; 0.035	-0.226	0.821
Tweets	-0.044	-0.069; -0.018	-3.386	0.001				
Tweets (WS)					-0.038	-0.065; -0.011	-2.730	0.006
Tweets (BS)					-0.101	-0.195; -0.007	-2.105	0.035
SDV	0.150	0.111; 0.189	7.455	<0.001	0.153	0.113; 0.194	7.400	<0.001
Tweets * SDV	0.006	-0.020; 0.032	0.461	0.645				
Tweets (WS) * SDV					0.002	-0.026; 0.030	0.134	0.894
Tweets (BS) * SDV					0.055	-0.043; 0.153	1.091	0.275

Notes. N = 1,341. Displays fixed-effect coefficients of multilevel models. All statistical tests were two-sided. The outcome of all models was the well-being on the consecutive day (lag-1). Degrees of freedom were >10,000 for all statistical tests. WS = within-subjects component; BS = between-

subjects component; b = unstandardized regression weight; 95%-CI = 95% confidence interval around the estimate; SDV = sociodemographic variable as indicated in the column head.

1.6. Results of supplementary analyses with the sub-facets of personality and well-being

Supplementary Table 16. Predicting well-being and its sub-facets by Stability and its sub-facets.

	Negative affect				Positive affect			
Predictors	b	95%-CI	*t*	*p*	b	95%-CI	*t*	*p*
(Intercept)	0.063	-0.005; 0.131	1.810	0.070	-0.081	-0.145; -0.017	-2.469	0.014
Level	0.178	0.096; 0.259	4.262	<0.001	-0.117	-0.190; -0.043	-3.108	0.002
Pre-event	0.007	-0.093; 0.106	0.132	0.895	0.068	-0.044; 0.180	1.185	0.236
Post-event	-0.067	-0.162; 0.028	-1.388	0.165	0.128	0.035; 0.222	2.690	0.007
Stability	-0.217	-0.286; -0.148	-6.150	<0.001	0.202	0.137; 0.267	6.109	<0.001
Level * Stability	-0.002	-0.080; 0.076	-0.055	0.956	-0.040	-0.110; 0.031	-1.106	0.269
Pre-event * Stability	0.060	-0.034; 0.154	1.255	0.210	-0.020	-0.126; 0.087	-0.362	0.718
Post-event * Stability	-0.124	-0.222; -0.026	-2.488	0.013	0.147	0.050; 0.243	2.980	0.003

	Well-being			
Predictors	b	95%-CI	*t*	*p*
(Intercept)	-0.090	-0.154; -0.025	-2.714	0.007
Level	-0.182	-0.260; -0.103	-4.525	<0.001
Pre-event	0.041	-0.061; 0.143	0.781	0.435
Post-event	0.121	0.029; 0.212	2.571	0.010
Stability	0.245	0.180; 0.311	7.338	<0.001
Level * Stability	-0.027	-0.102; 0.048	-0.698	0.485
Pre-event * Stability	-0.043	-0.139; 0.054	-0.867	0.386
Post-event * Stability	0.161	0.066; 0.255	3.320	0.001

Supplementary Table 16. (continued)

	Negative affect				Positive affect			
Predictors	b	95%-CI	*t*	*p*	b	95%-CI	*t*	*p*
(Intercept)	0.064	-0.004; 0.133	1.846	0.065	-0.083	-0.147; -0.020	-2.564	0.010
Level	0.168	0.085; 0.250	3.985	<0.001	-0.122	-0.196; -0.048	-3.244	0.001
Pre-event	-0.003	-0.101; 0.095	-0.060	0.952	0.084	-0.027; 0.196	1.477	0.140
Post-event	-0.087	-0.182; 0.007	-1.807	0.071	0.149	0.055; 0.243	3.106	0.002
Neuroticism	0.209	0.139; 0.278	5.878	<0.001	-0.233	-0.297; -0.168	-7.065	<0.001
Level * Neuroticism	-0.014	-0.093; 0.066	-0.337	0.736	-0.006	-0.077; 0.065	-0.174	0.862
Pre-event * Neuroticism	-0.087	-0.180; 0.006	-1.843	0.065	0.044	-0.061; 0.149	0.818	0.413
Post-event * Neuroticism	0.152	0.054; 0.250	3.041	0.002	-0.122	-0.219; -0.025	-2.476	0.013

	Well-being			
Predictors	b	95%-CI	*t*	*p*
(Intercept)	-0.092	-0.157; -0.028	-2.805	0.005
Level	-0.180	-0.259; -0.101	-4.479	<0.001
Pre-event	0.055	-0.045; 0.156	1.082	0.279
Post-event	0.145	0.053; 0.237	3.095	0.002
Neuroticism	-0.259	-0.324; -0.193	-7.746	<0.001
Level * Neuroticism	0.006	-0.071; 0.082	0.148	0.882
Pre-event * Neuroticism	0.075	-0.020; 0.170	1.547	0.122
Post-event * Neuroticism	-0.162	-0.257; -0.067	-3.338	0.001

Supplementary Table 16. (continued)

	Negative affect				Positive affect			
Predictors	b	95%-CI	*t*	*p*	b	95%-CI	*t*	*p*
(Intercept)	0.066	-0.003; 0.134	1.879	0.060	-0.081	-0.146; -0.015	-2.417	0.016
Level	0.200	0.118; 0.282	4.789	<0.001	-0.126	-0.199; -0.053	-3.382	0.001
Pre-event	0.029	-0.059; 0.118	0.652	0.514	0.039	-0.073; 0.151	0.685	0.493
Post-event	-0.042	-0.138; 0.054	-0.855	0.392	0.101	0.005; 0.197	2.072	0.038
Concientiousness	-0.184	-0.255; -0.114	-5.161	<0.001	0.087	0.021; 0.154	2.571	0.010
Level * Conscientiousness	-0.064	-0.144; 0.017	-1.552	0.121	-0.042	-0.114; 0.030	-1.146	0.252
Pre-event * Conscientiousness	0.039	-0.043; 0.122	0.930	0.353	-0.016	-0.120; 0.089	-0.297	0.766
Post-event * Conscientiousness	0.043	-0.057; 0.143	0.845	0.398	0.049	-0.050; 0.148	0.968	0.333

	Well-being			
Predictors	b	95%-CI	*t*	*p*
(Intercept)	-0.091	-0.157; -0.026	-2.725	0.006
Level	-0.201	-0.278; -0.123	-5.058	<0.001
Pre-event	0.009	-0.093; 0.111	0.166	0.868
Post-event	0.090	-0.004; 0.184	1.871	0.061
Concientiousness	0.160	0.093; 0.227	4.667	<0.001
Level * Conscientiousness	0.010	-0.067; 0.087	0.256	0.798
Pre-event * Conscientiousness	-0.029	-0.124; 0.066	-0.601	0.548
Post-event * Conscientiousness	0.004	-0.094; 0.101	0.074	0.941

Supplementary Table 16. (continued)

	Negative affect				Positive affect			
Predictors	b	95%-CI	*t*	*p*	b	95%-CI	*t*	*p*
(Intercept)	0.063	-0.006; 0.132	1.785	0.074	-0.080	-0.145; -0.014	-2.394	0.017
Level	0.182	0.101; 0.263	4.407	<0.001	-0.118	-0.191; -0.044	-3.147	0.002
Pre-event	0.055	-0.045; 0.155	1.076	0.282	0.039	-0.074; 0.151	0.669	0.503
Post-event	-0.045	-0.141; 0.050	-0.930	0.353	0.109	0.014; 0.203	2.250	0.024
Agreeableness	-0.053	-0.124; 0.017	-1.483	0.138	0.095	0.029; 0.161	2.802	0.005
Level * Agreeableness	0.072	-0.010; 0.153	1.726	0.084	-0.087	-0.160; -0.013	-2.314	0.021
Pre-event * Agreeableness	-0.064	-0.162; 0.035	-1.266	0.206	0.070	-0.042; 0.181	1.226	0.220
Post-event * Agreeableness	-0.146	-0.243; -0.049	-2.945	0.003	0.136	0.040; 0.232	2.766	0.006

	Well-being			
Predictors	b	95%-CI	*t*	*p*
(Intercept)	-0.088	-0.154; -0.021	-2.587	0.010
Level	-0.186	-0.264; -0.107	-4.643	<0.001
Pre-event	0.001	-0.102; 0.104	0.016	0.987
Post-event	0.095	0.001; 0.188	1.991	0.046
Agreeableness	0.086	0.018; 0.153	2.483	0.013
Level * Agreeableness	-0.099	-0.178; -0.020	-2.463	0.014
Pre-event * Agreeableness	0.084	-0.017; 0.186	1.624	0.104
Post-event * Agreeableness	0.168	0.073; 0.263	3.461	0.001

Notes. N = 1,341. Displays fixed-effect coefficients of multilevel models. All statistical tests were two-sided. The outcome of each model is indicated in the column head. Degrees of freedom were >10,000 for all statistical tests. b = unstandardized regression weight; 95%-CI = 95% confidence interval around the estimate.

Supplementary Table 17. Predicting well-being and its sub-facets by Plasticity and its sub-facets.

	**Negative affect**				**Positive affect**			
Predictors	b	95%-CI	*t*	*p*	b	95%-CI	*t*	*p*
(Intercept)	0.065	-0.004; 0.135	1.853	0.064	-0.082	-0.147; -0.017	-2.483	0.013
Level	0.193	0.114; 0.273	4.754	<0.001	-0.131	-0.203; -0.058	-3.541	<0.001
Pre-event	0.049	-0.047; 0.146	1.006	0.314	0.037	-0.074; 0.148	0.650	0.515
Post-event	-0.042	-0.138; 0.054	-0.865	0.387	0.107	0.011; 0.202	2.191	0.028
Plasticity	-0.066	-0.136; 0.005	-1.832	0.067	0.142	0.076; 0.207	4.242	<0.001
Level * Plasticity	0.020	-0.057; 0.097	0.513	0.608	-0.038	-0.107; 0.032	-1.062	0.288
Pre-event * Plasticity	0.041	-0.051; 0.133	0.878	0.380	0.007	-0.099; 0.112	0.122	0.903
Post-event * Plasticity	-0.011	-0.110; 0.088	-0.216	0.829	0.025	-0.073; 0.123	0.503	0.615

	**Well-being**			
Predictors	b	95%-CI	*t*	*p*
(Intercept)	-0.091	-0.158; -0.025	-2.709	0.007
Level	-0.201	-0.278; -0.123	-5.069	<0.001
Pre-event	0.001	-0.100; 0.103	0.027	0.979
Post-event	0.094	-0.001; 0.188	1.948	0.051
Plasticity	0.125	0.058; 0.192	3.669	<0.001
Level * Plasticity	-0.031	-0.106; 0.043	-0.820	0.412
Pre-event * Plasticity	-0.016	-0.113; 0.080	-0.332	0.740
Post-event * Plasticity	0.018	-0.079; 0.115	0.363	0.717

Supplementary Table 17. (continued)

	Negative affect				Positive affect			
Predictors	b	95%-CI	*t*	*p*	b	95%-CI	*t*	*p*
(Intercept)	0.066	-0.003; 0.135	1.882	0.060	-0.083	-0.147; -0.018	-2.518	0.012
Level	0.193	0.112; 0.273	4.688	<0.001	-0.130	-0.203; -0.057	-3.510	<0.001
Pre-event	0.049	-0.050; 0.147	0.967	0.334	0.039	-0.072; 0.150	0.682	0.495
Post-event	-0.045	-0.141; 0.050	-0.930	0.353	0.109	0.014; 0.204	2.243	0.025
Extraversion	-0.129	-0.199; -0.059	-3.618	<0.001	0.199	0.134; 0.264	6.038	<0.001
Level * Extraversion	-0.011	-0.085; 0.063	-0.282	0.778	0.009	-0.057; 0.075	0.257	0.797
Pre-event * Extraversion	0.005	-0.083; 0.093	0.111	0.912	0.010	-0.089; 0.110	0.205	0.837
Post-event * Extraversion	-0.003	-0.104; 0.098	-0.056	0.955	0.020	-0.080; 0.120	0.395	0.693

	Well-being			
Predictors	b	95%-CI	*t*	*p*
(Intercept)	-0.092	-0.157; -0.026	-2.754	0.006
Level	-0.200	-0.278; -0.123	-5.055	<0.001
Pre-event	0.005	-0.096; 0.107	0.104	0.917
Post-event	0.096	0.003; 0.190	2.013	0.044
Extraversion	0.197	0.131; 0.263	5.856	<0.001
Level * Extraversion	0.016	-0.055; 0.087	0.437	0.662
Pre-event * Extraversion	0.005	-0.086; 0.095	0.098	0.922
Post-event * Extraversion	0.008	-0.090; 0.107	0.167	0.868

Supplementary Table 17. (continued)

	Negative affect				Positive affect			
Predictors	b	95%-CI	*t*	*p*	b	95%-CI	*t*	*p*
(Intercept)	0.064	-0.005; 0.134	1.819	0.069	-0.079	-0.144; -0.013	-2.350	0.019
Level	0.193	0.113; 0.273	4.709	<0.001	-0.126	-0.197; -0.054	-3.446	0.001
Pre-event	0.055	-0.044; 0.154	1.084	0.278	0.037	-0.075; 0.148	0.646	0.518
Post-event	-0.039	-0.136; 0.057	-0.805	0.421	0.100	0.004; 0.196	2.049	0.040
Openness	0.034	-0.036; 0.103	0.950	0.342	0.012	-0.053; 0.078	0.366	0.714
Level * Openness	0.048	-0.035; 0.130	1.126	0.260	-0.087	-0.162; -0.013	-2.299	0.022
Pre-event * Openness	0.056	-0.043; 0.154	1.109	0.267	0.014	-0.097; 0.125	0.240	0.810
Post-event * Openness	-0.025	-0.120; 0.070	-0.514	0.607	0.038	-0.057; 0.133	0.790	0.429

	Well-being			
Predictors	b	95%-CI	*t*	*p*
(Intercept)	-0.087	-0.153; -0.020	-2.555	0.011
Level	-0.198	-0.274; -0.122	-5.107	<0.001
Pre-event	0.004	-0.096; 0.103	0.074	0.941
Post-event	0.086	-0.009; 0.181	1.782	0.075
Openness	-0.012	-0.079; 0.054	-0.356	0.722
Level * Openness	-0.078	-0.157; 0.000	-1.959	0.050
Pre-event * Openness	-0.025	-0.124; 0.074	-0.503	0.615
Post-event * Openness	0.037	-0.057; 0.131	0.777	0.437

Notes. N = 1,341. Displays fixed-effect coefficients of multilevel models. All statistical tests were two-sided. The outcome of each model is indicated in the column head. Degrees of freedom were >10,000 for all statistical tests. b = unstandardized regression weight; 95%-CI = 95% confidence interval around the estimate.

1.7. Results of supplementary analyses with societal well-being as outcome

Supplementary Table 18. Coefficients and model fits of models representing different societal well-being trajectories.

	Model 1a				Model 1b			
Predictors	b	95%-CI	*t*	*p*	b	95%-CI	*t*	*p*
(Intercept)	0.017	-0.029; 0.064	0.729	0.466	0.050	-0.001; 0.100	1.933	0.053
Level					0.127	0.061; 0.193	3.764	<0.001
AIC	25792.1				25412.12			

	Model 1c				Model 1d			
Predictors	b	95%-CI	*t*	*p*	b	95%-CI	*t*	*p*
(Intercept)	-0.048	-0.105; 0.010	-1.614	0.107	-0.021	-0.087; 0.045	-0.631	0.528
Level					0.090	0.023; 0.156	2.643	0.008
Post-event	0.152	0.065; 0.240	3.413	0.001	0.137	0.047; 0.227	2.978	0.003
AIC	24936.74				24726.18			

	Model 2a				Model 2b			
Predictors	b	95%-CI	*t*	*p*	b	95%-CI	*t*	*p*
(Intercept)	-0.020	-0.072; 0.031	-0.774	0.439	-0.010	-0.069; 0.048	-0.345	0.730
Time	0.125	0.062; 0.188	3.877	<0.001	0.109	0.038; 0.181	3.011	0.003
Level					0.040	-0.040; 0.121	0.979	0.327
AIC	24807.2				24657.87			

	Model 2c				Model 2d			
Predictors	b	95%-CI	*t*	*p*	b	95%-CI	*t*	*p*
(Intercept)	-0.020	-0.085; 0.044	-0.621	0.534	-0.010	-0.076; 0.057	-0.282	0.778
Level					0.051	-0.024; 0.126	1.333	0.182
Pre-event	0.160	0.052; 0.268	2.904	0.004	0.112	-0.007; 0.231	1.849	0.064
Post-event	0.136	0.045; 0.227	2.945	0.003	0.126	0.035; 0.217	2.714	0.007
AIC	24602.47				24489.15			

Notes. *N* = 1,280. Displays fixed-effect coefficients of multilevel models. All statistical tests were two-sided. The outcome of all models was societal well-being. The degrees of freedom were >10,000 for all statistical tests. b = unstandardized regression weight; 95%-CI = 95% confidence interval around the estimate.

Supplementary Table 19. Coefficients of Model 2d including additional level-2 variables predicting societal well-being.

	Model 2d				Model 2d + Stability			
Predictors	b	95%-CI	*t*	*p*	b	95%-CI	*t*	*p*
(Intercept)	-0.010	-0.076; 0.057	-0.282	0.778	-0.006	-0.073; 0.061	-0.179	0.858
Level	0.051	-0.024; 0.126	1.333	0.183	0.080	0.004; 0.155	2.064	0.039
Pre-event	0.112	-0.007; 0.231	1.849	0.064	0.141	0.021; 0.261	2.297	0.022
Post-event	0.126	0.035; 0.217	2.714	0.007	0.134	0.043; 0.225	2.890	0.004
Stability					0.099	0.031; 0.167	2.865	0.004
Level * Stability					-0.066	-0.137; 0.004	-1.852	0.064
Pre-event * Stability					-0.057	-0.170; 0.055	-1.001	0.317
Post-event * Stability					0.060	-0.034; 0.155	1.246	0.213

	Model 2d + Age				Model 2d + Gender			
Predictors	b	95%-CI	*t*	*p*	b	95%-CI	*t*	*p*
(Intercept)	-0.022	-0.088; 0.045	-0.634	0.526	0.022	-0.052; 0.096	0.584	0.559
Level	0.035	-0.041; 0.112	0.908	0.364	0.040	-0.043; 0.123	0.945	0.345
Pre-event	0.106	-0.014; 0.226	1.726	0.084	0.134	0.005; 0.264	2.033	0.042
Post-event	0.136	0.045; 0.228	2.926	0.003	0.155	0.054; 0.255	3.004	0.003
SDV	-0.133	-0.205; -0.062	-3.650	<0.001	-0.144	-0.316; 0.029	-1.635	0.102
Level * SDV	0.042	-0.033; 0.118	1.099	0.272	0.074	-0.118; 0.267	0.758	0.449
Pre-event * SDV	-0.159	-0.275; -0.044	-2.711	0.007	-0.109	-0.432; 0.213	-0.664	0.507
Post-event * SDV	0.127	0.030; 0.225	2.553	0.011	-0.171	-0.406; 0.064	-1.428	0.153

	Model 2d + Political attitude				Model 2d + Social status			
Predictors	b	95%-CI	*t*	*p*	b	95%-CI	*t*	*p*
(Intercept)	-0.009	-0.076; 0.057	-0.278	0.781	-0.009	-0.076; 0.058	-0.258	0.796
Level	0.046	-0.029; 0.121	1.202	0.229	0.055	-0.020; 0.130	1.432	0.152
Pre-event	0.118	0.000; 0.235	1.962	0.050	0.142	0.022; 0.263	2.312	0.021
Post-event	0.126	0.035; 0.217	2.708	0.007	0.127	0.036; 0.218	2.725	0.006
SDV	0.027	-0.041; 0.095	0.780	0.436	0.047	-0.021; 0.114	1.363	0.173
Level * SDV	-0.035	-0.111; 0.040	-0.916	0.360	-0.003	-0.078; 0.071	-0.086	0.932
Pre-event * SDV	-0.103	-0.223; 0.016	-1.694	0.090	-0.096	-0.217; 0.025	-1.554	0.120
Post-event * SDV	-0.005	-0.098; 0.087	-0.113	0.910	-0.024	-0.116; 0.068	-0.511	0.610

Notes. N = 1,280. Displays fixed-effect coefficients of multilevel models. All statistical tests were two-sided. The outcome of all models was societal well-being. Degrees of freedom were >10,000

for all statistical tests. b = unstandardized regression weight; 95%-CI = 95% confidence interval around the estimate; SDV = sociodemographic variable as indicated in the column head.

Supplementary Table 20. Predicting societal well-being by the salience of the war and additional level-2 predictors.

	**Salience**				**Salience (WS/BS)**			
Predictors	b	95%-CI	*t*	*p*	b	95%-CI	*t*	*p*
(Intercept)	0.016	-0.031; 0.063	0.670	0.503	0.017	-0.031; 0.065	0.689	0.491
Tweets	-0.020	-0.044; 0.003	-1.737	0.082				
Tweets (WS)					-0.024	-0.048; 0.000	-1.959	0.050
Tweets (BS)					-0.010	-0.119; 0.100	-0.172	0.864

	**Salience + Stability**				**Salience (WS/BS) + Stability**			
Predictors	b	95%-CI	*t*	*p*	b	95%-CI	*t*	*p*
(Intercept)	0.015	-0.031; 0.061	0.624	0.533	0.016	-0.031; 0.063	0.670	0.503
Tweets	-0.017	-0.040; 0.007	-1.417	0.156				
Tweets (WS)					-0.022	-0.047; 0.002	-1.764	0.078
Tweets (BS)					-0.001	-0.110; 0.107	-0.027	0.979
Stability	0.145	0.099; 0.192	6.151	<0.001	0.150	0.102; 0.198	6.164	<0.001
Tweets * Stability	-0.013	-0.036; 0.009	-1.201	0.230				
Tweets (WS) * Stability					-0.013	-0.036; 0.010	-1.123	0.262
Tweets (BS) * Stability					0.017	-0.092; 0.125	0.302	0.763

	**Salience + Age**				**Salience (WS/BS) + Age**			
Predictors	b	95%-CI	*t*	*p*	b	95%-CI	*t*	*p*
(Intercept)	0.016	-0.031; 0.062	0.655	0.512	0.015	-0.033; 0.063	0.607	0.544
Tweets	-0.021	-0.045; 0.003	-1.721	0.085				
Tweets (WS)					-0.024	-0.049; 0.001	-1.887	0.059
Tweets (BS)					-0.018	-0.129; 0.093	-0.319	0.750
SDV	-0.070	-0.116; -0.023	-2.943	0.003	-0.075	-0.126; -0.023	-2.855	0.004
Tweets * SDV	0.000	-0.023; 0.023	0.011	0.991				
Tweets (WS) * SDV					0.003	-0.021; 0.027	0.270	0.787
Tweets (BS) * SDV					-0.030	-0.139; 0.078	-0.550	0.582

Supplementary Table 20. (continued)

	Salience + Gender				Salience (WS/BS) + Gender			
Predictors	b	95%-CI	*t*	*p*	b	95%-CI	*t*	*p*
(Intercept)	0.060	0.008; 0.111	2.267	0.023	0.059	0.006; 0.112	2.198	0.028
Tweets	-0.026	-0.051; -0.000	-1.970	0.049				
Tweets (WS)					-0.029	-0.055; -0.002	-2.120	0.034
Tweets (BS)					-0.028	-0.145; 0.090	-0.461	0.645
SDV	-0.228	-0.349; -0.107	-3.692	<0.001	-0.220	-0.344; -0.095	-3.466	0.001
Tweets * SDV	0.031	-0.029; 0.091	1.013	0.311				
Tweets (WS) * SDV					0.027	-0.035; 0.089	0.861	0.389
Tweets (BS) * SDV					0.136	-0.182; 0.453	0.838	0.402

	Salience + Political attitude				Salience (WS/BS) + Political attitude			
Predictors	b	95%-CI	*t*	*p*	b	95%-CI	*t*	*p*
(Intercept)	0.016	-0.031; 0.063	0.675	0.499	0.018	-0.030; 0.066	0.745	0.456
Tweets	-0.021	-0.044; 0.003	-1.733	0.083				
Tweets (WS)					-0.025	-0.049; -0.000	-1.985	0.047
Tweets (BS)					-0.000	-0.112; 0.111	-0.002	0.999
SDV	0.053	0.006; 0.100	2.206	0.027	0.056	0.007; 0.105	2.238	0.025
Tweets * SDV	-0.007	-0.031; 0.017	-0.585	0.559				
Tweets (WS) * SDV					-0.007	-0.032; 0.018	-0.575	0.565
Tweets (BS) * SDV					0.013	-0.105; 0.131	0.216	0.829

	Salience + Social status				Salience (WS/BS) + Social status			
Predictors	b	95%-CI	*t*	*p*	b	95%-CI	*t*	*p*
(Intercept)	0.016	-0.030; 0.063	0.690	0.490	0.018	-0.030; 0.065	0.720	0.471
Tweets	-0.021	-0.044; 0.003	-1.740	0.082				
Tweets (WS)					-0.025	-0.049; -0.001	-2.004	0.045
Tweets (BS)					-0.005	-0.116; 0.106	-0.086	0.932
SDV	0.037	-0.010; 0.084	1.545	0.122	0.038	-0.011; 0.086	1.516	0.129
Tweets * SDV	0.001	-0.022; 0.025	0.114	0.910				
Tweets (WS) * SDV					0.003	-0.022; 0.027	0.214	0.831
Tweets (BS) * SDV					-0.003	-0.117; 0.111	-0.057	0.954

Notes. N = 1,280. Displays fixed-effect coefficients of multilevel models. All statistical tests were two-sided. The outcome of all models was societal well-being. Degrees of freedom were >10,000 for all statistical tests. WS = within-subjects component; BS = between-subjects component; b =

unstandardized regression weight; 95%-CI = 95% confidence interval around the estimate; SDV = sociodemographic variable as indicated in the column head.

1.8. Results of analyses with Ukraine-related variables

Supplementary Table 21. Predicting well-being by Ukraine-related variables.

Predictors		Individual well-being			
		b	95%-CI	*t*	*p*
Ukraine-related behavior 1	*"How much news do you consume in comparison to before the war in Ukraine?" (higher values = more news)*	0.033	-0.024; 0.089	1.135	0.257
Ukraine-related behavior 2	*"I show my solidarity with Ukraine (for example through donations, participation in protests or public positioning)."*	0.040	-0.017; 0.096	1.382	0.167
Ukraine-related behavior 3	*"I purposefully reduce my news consumption concerning the war in Ukraine."*	0.044	-0.012; 0.101	1.535	0.125
Ukraine-related behavior 4	*"I prepare myself for further escalation of the conflict by keeping vital goods in stock (e.g., water, toilet paper, canned products, or petrol)."*	-0.120	-0.176; -0.064	-4.235	<0.001
Ukraine-related emotion 1	*"I am worried about my physical well-being due to the war in Ukraine."*	-0.119	-0.175; -0.063	-4.185	<0.001
Ukraine-related emotion 2	*"I am worried about the physical well-being of my family due to the war in Ukraine."*	-0.109	-0.165; -0.053	-3.840	<0.001
Ukraine-related emotion 3	*"I am worried about my psychological well-being due to the war in Ukraine."*	-0.142	-0.197; -0.086	-5.014	<0.001
Ukraine-related emotion 4	*"I am worried when I consume news about the war in Ukraine."*	-0.096	-0.153; -0.040	-3.362	0.001
Ukraine-related emotion 5	*"I am worried that the war will spread to other countries."*	-0.094	-0.150; -0.038	-3.304	0.001
Ukraine-related emotion 6	*"I am worried about the economic situation in my country."*	-0.100	-0.156; -0.043	-3.469	0.001
Ukraine-related emotion 7	*"I strongly feel for the affected people in the war zone."*	-0.074	-0.130; -0.017	-2.545	0.011
Ukraine-related evaluation 1	*"I assume that due to the war in Ukraine the third world war will come."*	-0.105	-0.161; -0.049	-3.696	<0.001
Ukraine-related evaluation 2	*"I consider the political proceedings of my country concerning the war in Ukraine ..." (higher values = stronger approval)*	0.107	0.051; 0.163	3.723	<0.001
Ukraine-related evaluation 3	*"I consider an increase in the defense budget in my country reasonable."*	0.036	-0.021; 0.092	1.235	0.217
Ukraine-related evaluation 4	*"I consider reporting of the public media in my country concerning the war in Ukraine factual."*	0.053	-0.003; 0.110	1.847	0.065
Brief COPE: problem-focused coping	active coping, use of informational support, planning, and positive reframing	0.098	0.042; 0.155	3.435	0.001
Brief COPE: emotion-focused coping	venting, use of emotional support, humor, acceptance, self-blame, and religion	0.075	0.018; 0.131	2.588	0.010
Brief COPE: avoidant coping	self-distraction, denial, substance use, and behavioral disengagement	-0.095	-0.151; -0.038	-3.292	0.001

Notes. n = 688 for the analyses including the Ukraine-related behaviors, emotions, and evaluations. *n* = 682 for the analyses including the Brief COPE. Displays fixed-effect coefficients of multilevel models. All statistical tests were two-sided. The degrees of freedom were >9,000 for all statistical tests. b = unstandardized regression weight; 95%-CI = 95% confidence interval around the estimate.

1.9. Results of analyses with Bayesian estimator

Supplementary Table 22. Coefficients and model fits of eight multilevel models representing different well-being trajectories.

	**Model 1a**		**Model 1b**		**Model 1c**	
Predictors	b	95%-CI	b	95%-CI	b	95%-CI
Intercept	0.003	-0.036; 0.042	-0.046	-0.089; -0.003	0.021	-0.030; 0.072
Level			-0.171	-0.230; -0.112		
Post-event					-0.060	-0.139; 0.020

	**Model 1d**		**Model 2a**		**Model 2b**	
Predictors	b	95%-CI	b	95%-CI	b	95%-CI
Intercept	-0.094	-0.160; -0.028	0.018	-0.025; 0.060	-0.075	-0.130; -0.021
Time			-0.062	-0.115; -0.010	0.056	-0.015; 0.126
Level	-0.202	-0.273; -0.132			-0.205	-0.286; -0.125
Post-event	0.093	-0.001; 0.186				

	**Model 2c**		**Model 2d**	
Predictors	b	95%-CI	b	95%-CI
Intercept	-0.021	-0.080; 0.038	-0.091	-0.156; -0.024
Level			-0.203	-0.284; -0.123
Pre-event	-0.195	-0.297; -0.094	0.008	-0.097; 0.112
Post-event	-0.008	-0.093; 0.077	0.090	-0.005; 0.184

Notes. N = 1,341. Displays fixed-effect coefficients of Bayesian multilevel models. The outcome of all models was well-being. b = unstandardized regression weight; 95%-CI = 95% credible interval around the estimate.

Supplementary Table 23. Coefficients of Model 2d including additional level-2 predictors.

	Model 2d		Model 2d + Stability	
Predictors	b	95%-CI	b	95%-CI
Intercept	-0.091	-0.156; -0.024	-0.090	-0.155; -0.026
Level	-0.203	-0.284; -0.123	-0.183	-0.263; -0.103
Pre-event	0.008	-0.097; 0.112	0.041	-0.063; 0.144
Post-event	0.090	-0.005; 0.184	0.122	0.031; 0.213
Stability			0.245	0.180; 0.311
Level * Stability			-0.026	-0.103; 0.049
Pre-event * Stability			-0.042	-0.141; 0.056
Post-event * Stability			0.160	0.065; 0.254

	Model 2d + Age		Model 2d + Gender	
Predictors	b	95%-CI	b	95%-CI
Intercept	-0.096	-0.163; -0.028	-0.116	-0.190; -0.042
Level	-0.190	-0.271; -0.111	-0.212	-0.301; -0.123
Pre-event	0.026	-0.076; 0.126	0.003	-0.113; 0.114
Post-event	0.107	0.011; 0.203	0.075	-0.029; 0.180
SDV	0.002	-0.071; 0.074	0.125	-0.048; 0.297
Level * SDV	-0.020	-0.105; 0.064	0.047	-0.156; 0.251
Pre-event * SDV	-0.127	-0.231; -0.019	-0.043	-0.328; 0.241
Post-event * SDV	0.071	-0.033; 0.175	0.081	-0.163; 0.326

	Model 2d + Political attitude		Model 2d+ Social status	
Predictors	b	95%-CI	b	95%-CI
Intercept	-0.092	-0.158; -0.025	-0.090	-0.156; -0.024
Level	-0.209	-0.288; -0.129	-0.200	-0.280; -0.122
Pre-event	0.011	-0.095; 0.114	0.028	-0.079; 0.132
Post-event	0.090	-0.004; 0.185	0.108	0.014; 0.203
SDV	0.010	-0.058; 0.077	0.100	0.032; 0.167
Level * SDV	-0.053	-0.133; 0.026	0.012	-0.070; 0.093
Pre-event * SDV	-0.001	-0.107; 0.106	-0.017	-0.126; 0.093
Post-event * SDV	0.032	-0.063; 0.128	0.082	-0.015; 0.180

Notes. N = 1,341. Displays fixed-effect coefficients of Bayesian multilevel models. The outcome of all models was well-being. b = unstandardized regression weight; 95%-CI = 95% credible interval around the estimate; SDV = sociodemographic variable as indicated in the column head.

Supplementary Table 24. Predicting well-being by the salience of the war and additional level-2 predictors.

	Salience		Salience (WS/BS)	
Predictors	b	95%-CI	b	95%-CI
Intercept	-0.004	-0.044; 0.035	-0.007	-0.046; 0.033
Tweets	-0.070	-0.097; -0.044		
Tweets (WS)			-0.065	-0.093; -0.036
Tweets (BS)			-0.116	-0.209; -0.022

	Salience + Stability		Salience (WS/BS) +Stability	
Predictors	b	95%-CI	b	95%-CI
Intercept	-0.003	-0.038; 0.032	-0.006	-0.042; 0.029
Tweets	-0.071	-0.097; -0.044		
Tweets (WS)			-0.067	-0.095; -0.038
Tweets (BS)			-0.101	-0.184; -0.017
Stability	0.335	0.299; 0.370	0.334	0.297; 0.370
Tweets * Stability	-0.013	-0.038; 0.012		
Tweets (WS) * Stability			-0.010	-0.038; 0.017
Tweets (BS) * Stability			-0.038	-0.119; 0.044

	Salience + Age		Salience (WS/BS) + Age	
Predictors	b	95%-CI	b	95%-CI
Intercept	-0.001	-0.040; 0.038	-0.004	-0.044; 0.035
Tweets	-0.069	-0.095; -0.042		
Tweets (WS)			-0.064	-0.092; -0.034
Tweets (BS)			-0.108	-0.199; -0.013
SDV	0.082	0.042; 0.123	0.089	0.045; 0.133
Tweets * SDV	-0.010	-0.037; 0.017		
Tweets (WS) * SDV			-0.012	-0.042; 0.017
Tweets (BS) * SDV			0.022	-0.070; 0.113

Supplementary Table 24. (continued)

	Salience + Gender		Salience (WS/BS) +. Gender	
Predictors	b	95%-CI	b	95%-CI
Intercept	-0.032	-0.076; 0.012	-0.038	-0.083; 0.007
Tweets	-0.068	-0.097; -0.039		
Tweets (WS)			-0.059	-0.090; -0.026
Tweets (BS)			-0.141	-0.243; -0.039
SDV	0.150	0.050; 0.249	0.160	0.057; 0.264
Tweets * SDV	-0.017	-0.086; 0.054		
Tweets (WS) * SDV			-0.030	-0.104; 0.043
Tweets (BS) * SDV			0.111	-0.132; 0.354

	Salience + Political attitude		Salience (WS/BS) + Political attitude	
Predictors	b	95%-CI	b	95%-CI
Intercept	-0.004	-0.043; 0.035	-0.007	-0.047; 0.033
Tweets	-0.072	-0.098; -0.045		
Tweets (WS)			-0.067	-0.095; -0.038
Tweets (BS)			-0.110	-0.204; -0.017
SDV	0.036	-0.003; 0.075	0.039	-0.001; 0.079
Tweets * SDV	-0.013	-0.041; 0.015		
Tweets (WS) * SDV			-0.014	-0.044; 0.015
Tweets (BS) * SDV			0.009	-0.089; 0.106

	Salience + Social status		Salience (WS/BS) + Social status	
Predictors	b	95%-CI	b	95%-CI
Intercept	-0.002	-0.040; 0.037	-0.004	-0.043; 0.035
Tweets	-0.070	-0.096; -0.044		
Tweets (WS)			-0.066	-0.093; -0.037
Tweets (BS)			-0.102	-0.194; -0.011
SDV	0.149	0.109; 0.188	0.150	0.110; 0.190
Tweets * SDV	0.003	-0.025; 0.030		
Tweets (WS) * SDV			0.000	-0.029; 0.030
Tweets (BS) * SDV			0.030	-0.066; 0.123

Notes. N = 1,341. Displays fixed-effect coefficients of Bayesian multilevel models. The outcome of all models was well-being. WS = within-subjects component; BS = between-subjects component; b = unstandardized regression weight; 95%-CI = 95% credible interval around the estimate; SDV = sociodemographic variable as indicated in the column head.

Supplementary Table 25. Coefficients of Model 2d including country variables.

	**Model 2d**		**Model 2d + Stability**	
Predictors	b	95%-CI	b	95%-CI
Intercept	0.136	-0.197; 0.473	0.067	-0.237; 0.367
Level	-0.164	-0.245; -0.081	-0.163	-0.245; -0.078
Pre-event	0.068	-0.038; 0.171	0.084	-0.021; 0.189
Post-event	0.097	0.001; 0.195	0.101	0.006; 0.194
Stability			0.235	0.168; 0.302
Level * Stability			-0.034	-0.112; 0.042
Pre-event * Stability			-0.048	-0.145; 0.049
Post-event * Stability			0.148	0.053; 0.243
France	0.325	-0.076; 0.710	0.293	-0.058; 0.651
Germany	0.001	-0.343; 0.342	-0.080	-0.385; 0.229
Italy	-0.343	-0.680; -0.009	-0.200	-0.501; 0.107
Netherlands	-0.125	-0.566; 0.321	-0.131	-0.527; 0.267
Poland	-0.230	-0.587; 0.123	-0.148	-0.461; 0.173
Turkey	-0.251	-0.605; 0.099	-0.254	-0.571; 0.067
United Kingdom	-0.088	-0.440; 0.257	0.010	-0.302; 0.324

Supplementary Table 25. (continued)

	Model 2d + Age		Model 2d + Gender	
Predictors	b	95%-CI	b	95%-CI
Intercept	0.119	-0.214; 0.449	0.119	-0.227; 0.462
Level	-0.147	-0.228; -0.063	-0.170	-0.259; -0.080
Pre-event	0.082	-0.020; 0.184	0.070	-0.045; 0.182
Post-event	0.106	0.008; 0.203	0.080	-0.026; 0.190
SDV	-0.029	-0.105; 0.046	0.131	-0.040; 0.303
Level * SDV	-0.052	-0.136; 0.033	0.045	-0.157; 0.251
Pre-event * SDV	-0.119	-0.225; -0.013	-0.060	-0.343; 0.227
Post-event * SDV	0.068	-0.035; 0.172	0.101	-0.142; 0.345
France	0.331	-0.055; 0.722	0.319	-0.079; 0.719
Germany	-0.020	-0.357; 0.317	0.007	-0.347; 0.363
Italy	-0.328	-0.658; 0.007	-0.359	-0.702; -0.012
Netherlands	-0.126	-0.558; 0.314	-0.136	-0.585; 0.319
Poland	-0.236	-0.587; 0.113	-0.234	-0.597; 0.129
Turkey	-0.234	-0.586; 0.116	-0.263	-0.623; 0.099
United Kingdom	-0.018	-0.364; 0.326	-0.083	-0.444; 0.275

Supplementary Table 25. (continued)

	**Model 2d + Political attitude**		**Model 2d + Social status**	
Predictors	b	95%-CI	b	95%-CI
Intercept	0.140	-0.195; 0.471	0.143	-0.190; 0.476
Level	-0.168	-0.248; -0.085	-0.168	-0.250; -0.086
Pre-event	0.071	-0.033; 0.176	0.077	-0.032; 0.184
Post-event	0.098	0.001; 0.197	0.110	0.013; 0.209
SDV	0.017	-0.051; 0.086	0.076	0.007; 0.145
Level * SDV	-0.045	-0.125; 0.037	0.008	-0.073; 0.090
Pre-event * SDV	0.002	-0.104; 0.106	-0.014	-0.124; 0.095
Post-event * SDV	0.033	-0.063; 0.129	0.089	-0.006; 0.185
France	0.319	-0.075; 0.709	0.291	-0.098; 0.676
Germany	0.003	-0.340; 0.343	-0.039	-0.383; 0.301
Italy	-0.350	-0.683; -0.014	-0.334	-0.670; 0.003
Netherlands	-0.119	-0.554; 0.319	-0.182	-0.622; 0.256
Poland	-0.226	-0.577; 0.128	-0.238	-0.592; 0.112
Turkey	-0.249	-0.606; 0.101	-0.259	-0.615; 0.091
United Kingdom	-0.094	-0.443; 0.253	-0.099	-0.448; 0.250

Notes. N = 1,341. Displays fixed-effect coefficients of Bayesian multilevel models. The outcome of all models was well-being. b = unstandardized regression weight; 95%-CI = 95% credible interval around the estimate; SDV = sociodemographic variable as indicated in the column head.

Supplementary Table 26. Predicting well-being by the salience of the war, country, and additional level-2 predictors.

	Salience		Salience (WS/BS)	
Predictors	b	95%-CI	b	95%-CI
Intercept	0.227	-0.106; 0.556	0.246	-0.070; 0.565
Tweets	-0.068	-0.094; -0.041		
Tweets (WS)			-0.070	-0.097; -0.042
Tweets (BS)			-0.044	-0.137; 0.049
France	0.290	-0.098; 0.673	0.272	-0.101; 0.642
Germany	-0.001	-0.345; 0.341	-0.020	-0.354; 0.308
Italy	-0.379	-0.713; -0.036	-0.396	-0.723; -0.076
Netherlands	-0.136	-0.575; 0.304	-0.164	-0.600; 0.270
Poland	-0.253	-0.612; 0.102	-0.282	-0.630; 0.066
Turkey	-0.258	-0.613; 0.101	-0.285	-0.632; 0.058
United Kingdom	-0.082	-0.435; 0.270	-0.094	-0.434; 0.242

	Salience + Stability		Salience (WS/BS) + Stability	
Predictors	b	95%-CI	b	95%-CI
Intercept	0.152	-0.141; 0.456	0.160	-0.137; 0.455
Tweets	-0.065	-0.091; -0.037		
Tweets (WS)			-0.068	-0.096; -0.039
Tweets (BS)			-0.031	-0.117; 0.055
Stability	0.318	0.282; 0.354	0.318	0.281; 0.355
Tweets * Stability	-0.013	-0.039; 0.012		
Tweets (WS) * Stability			-0.010	-0.037; 0.017
Tweets (BS) * Stability			-0.044	-0.124; 0.036
France	0.279	-0.071; 0.622	0.275	-0.064; 0.623
Germany	-0.085	-0.403; 0.219	-0.095	-0.400; 0.210
Italy	-0.229	-0.536; 0.065	-0.237	-0.535; 0.064
Netherlands	-0.151	-0.556; 0.246	-0.159	-0.551; 0.238
Poland	-0.167	-0.494; 0.150	-0.193	-0.511; 0.132
Turkey	-0.267	-0.595; 0.051	-0.279	-0.597; 0.043
United Kingdom	0.010	-0.312; 0.321	0.017	-0.297; 0.330

Supplementary Table 26. (continued)

	Salience + Age		Salience (WS/BS) + Age	
Predictors	b	95%-CI	b	95%-CI
Intercept	0.207	-0.123; 0.537	0.228	-0.089; 0.546
Tweets	-0.064	-0.091; -0.036		
Tweets (WS)			-0.068	-0.096; -0.038
Tweets (BS)			-0.023	-0.122; 0.074
SDV	0.050	0.008; 0.092	0.056	0.011; 0.101
Tweets * SDV	-0.011	-0.038; 0.016		
Tweets (WS) * SDV			-0.009	-0.039; 0.020
Tweets (BS) * SDV			-0.014	-0.108; 0.079
France	0.309	-0.073; 0.689	0.292	-0.080; 0.664
Germany	-0.013	-0.358; 0.335	-0.036	-0.366; 0.298
Italy	-0.343	-0.679; -0.007	-0.361	-0.685; -0.037
Netherlands	-0.149	-0.584; 0.292	-0.176	-0.614; 0.256
Poland	-0.255	-0.610; 0.105	-0.290	-0.635; 0.055
Turkey	-0.253	-0.603; 0.105	-0.285	-0.632; 0.054
United Kingdom	-0.036	-0.388; 0.318	-0.035	-0.383; 0.308

Supplementary Table 26. (continued)

	**Salience + Gender**		**Salience (WS/BS) + Gender**	
Predictors	b	95%-CI	b	95%-CI
Intercept	0.200	-0.134; 0.534	0.215	-0.118; 0.544
Tweets	-0.063	-0.093; -0.034		
Tweets (WS)			-0.064	-0.095; -0.033
Tweets (BS)			-0.054	-0.155; 0.047
SDV	0.172	0.075; 0.271	0.178	0.078; 0.278
Tweets * SDV	-0.026	-0.094; 0.044		
Tweets (WS) * SDV			-0.031	-0.104; 0.042
Tweets (BS) * SDV			0.029	-0.209; 0.271
France	0.289	-0.091; 0.672	0.276	-0.108; 0.660
Germany	0.011	-0.335; 0.352	-0.009	-0.350; 0.337
Italy	-0.390	-0.728; -0.053	-0.404	-0.735; -0.068
Netherlands	-0.143	-0.587; 0.303	-0.161	-0.614; 0.291
Poland	-0.251	-0.615; 0.106	-0.277	-0.635; 0.087
Turkey	-0.272	-0.629; 0.086	-0.297	-0.651; 0.062
United Kingdom	-0.073	-0.424; 0.280	-0.083	-0.432; 0.271

Supplementary Table 26. (continued)

	**Salience + Political attitude**		**Salience (WS/BS) + Political attitude**	
Predictors	b	95%-CI	b	95%-CI
Intercept	0.244	-0.083; 0.570	0.261	-0.064; 0.588
Tweets	-0.069	-0.095; -0.042		
Tweets (WS)			-0.072	-0.100; -0.044
Tweets (BS)			-0.035	-0.131; 0.060
SDV	0.043	0.004; 0.081	0.048	0.008; 0.087
Tweets * SDV	-0.012	-0.040; 0.015		
Tweets (WS) * SDV			-0.016	-0.045; 0.013
Tweets (BS) * SDV			0.021	-0.073; 0.116
France	0.269	-0.108; 0.644	0.256	-0.124; 0.628
Germany	-0.015	-0.355; 0.324	-0.029	-0.368; 0.306
Italy	-0.396	-0.732; -0.066	-0.413	-0.747; -0.084
Netherlands	-0.147	-0.583; 0.288	-0.173	-0.611; 0.267
Poland	-0.263	-0.616; 0.092	-0.291	-0.646; 0.062
Turkey	-0.271	-0.625; 0.084	-0.296	-0.646; 0.056
United Kingdom	-0.102	-0.451; 0.245	-0.108	-0.451; 0.238

Supplementary Table 26. (continued)

	Salience + Social status		Salience (WS/BS) + Social status	
Predictors	b	95%-CI	b	95%-CI
Intercept	0.243	-0.088; 0.557	0.262	-0.054; 0.584
Tweets	-0.067	-0.093; -0.040		
Tweets (WS)			-0.070	-0.098; -0.041
Tweets (BS)			-0.037	-0.130; 0.057
SDV	0.123	0.084; 0.162	0.127	0.086; 0.167
Tweets * SDV	0.004	-0.023; 0.031		
Tweets (WS) * SDV			0.001	-0.028; 0.030
Tweets (BS) * SDV			0.037	-0.058; 0.132
France	0.262	-0.103; 0.641	0.244	-0.131; 0.608
Germany	-0.061	-0.390; 0.281	-0.078	-0.415; 0.250
Italy	-0.371	-0.688; -0.037	-0.388	-0.715; -0.069
Netherlands	-0.217	-0.641; 0.221	-0.250	-0.684; 0.185
Poland	-0.266	-0.613; 0.089	-0.295	-0.646; 0.044
Turkey	-0.280	-0.619; 0.075	-0.307	-0.655; 0.033
United Kingdom	-0.099	-0.433; 0.247	-0.109	-0.453; 0.227

Notes. *N* = 1,341. Displays fixed-effect coefficients of Bayesian multilevel models. The outcome of all models was well-being. WS = within-subjects component; BS = between-subjects component; b = unstandardized regression weight; 95%-CI = 95% credible interval around the estimate; SDV = sociodemographic variable as indicated in the column head.

Supplementary Table 27. Coefficients and model fits of models representing different well-being trajectories excluding one country.

	Without France (*N* = 1,280)		Without Germany (*N* = 1,122)		Without Italy (*N* = 682)	
Predictors	b	95%-CI	b	95%-CI	b	95%-CI
Intercept	-0.101	-0.168; -0.033	-0.122	-0.196; -0.050	-0.077	-0.168; 0.015
Level	-0.203	-0.283; -0.123	-0.193	-0.287; -0.100	-0.249	-0.337; -0.161
Pre-event	0.001	-0.105; 0.105	0.009	-0.123; 0.137	0.045	-0.061; 0.147
Post-event	0.062	-0.035; 0.159	0.083	-0.023; 0.188	0.422	0.279; 0.564

	Without the Netherlands (*N* = 1,319)		Without Poland (*N* = 1,238)		Without Turkey (*N* = 1,229)	
Predictors	b	95%-CI	b	95%-CI	b	95%-CI
Intercept	-0.089	-0.156; -0.021	-0.060	-0.130; 0.011	-0.087	-0.160; -0.015
Level	-0.194	-0.274; -0.113	-0.187	-0.273; -0.102	-0.277	-0.371; -0.184
Pre-event	0.002	-0.105; 0.105	0.041	-0.074; 0.150	0.058	-0.051; 0.165
Post-event	0.086	-0.008; 0.180	0.051	-0.047; 0.149	0.067	-0.031; 0.165

	Without the United Kingdom (*N* = 1,196)	
Predictors	b	95%-CI
Intercept	-0.110	-0.177; -0.040
Level	-0.153	-0.237; -0.068
Pre-event	-0.120	-0.254; 0.011
Post-event	0.096	-0.003; 0.195

Notes. Displays fixed-effect coefficients of Bayesian multilevel models. The outcome of all models was well-being. b = unstandardized regression weight; 95%-CI = 95% credible interval around the estimate.

Supplementary Table 28. Coefficients of Model 2d including additional level-2 predictors excluding one country.

	**Without France** (*N* = 1,280)		**Without Germany** (*N* = 1,122)		**Without Italy** (*N* = 682)	
Predictors	b	95%-CI	b	95%-CI	b	95%-CI
Intercept	-0.098	-0.162; -0.033	-0.102	-0.172; -0.030	-0.146	-0.237; -0.056
Level	-0.184	-0.264; -0.102	-0.179	-0.271; -0.085	-0.258	-0.347; -0.169
Pre-event	0.036	-0.069; 0.139	0.009	-0.123; 0.136	0.055	-0.049; 0.159
Post-event	0.100	0.006; 0.193	0.124	0.024; 0.226	0.431	0.289; 0.575
Stability	0.242	0.176; 0.307	0.247	0.176; 0.319	0.302	0.217; 0.386
Level * Stability	-0.027	-0.105; 0.048	-0.005	-0.095; 0.081	0.020	-0.064; 0.102
Pre-event * Stability	-0.044	-0.143; 0.053	-0.054	-0.177; 0.067	-0.030	-0.129; 0.066
Post-event * Stability	0.153	0.057; 0.250	0.169	0.063; 0.273	-0.064	-0.201; 0.076

	**Without the Netherlands** (*N* = 1,319)		**Without Poland** (*N* = 1,238)		**Without Turkey** (*N* = 1,229)	
Predictors	b	95%-CI	b	95%-CI	b	95%-CI
Intercept	-0.087	-0.152; -0.021	-0.062	-0.130; 0.006	-0.074	-0.146; -0.003
Level	-0.174	-0.255; -0.093	-0.161	-0.246; -0.076	-0.243	-0.337; -0.149
Pre-event	0.038	-0.069; 0.143	0.065	-0.045; 0.173	0.094	-0.015; 0.200
Post-event	0.118	0.026; 0.210	0.084	-0.011; 0.178	0.092	-0.003; 0.189
Stability	0.245	0.178; 0.310	0.250	0.181; 0.319	0.229	0.154; 0.302
Level * Stability	-0.019	-0.098; 0.057	-0.037	-0.122; 0.046	-0.065	-0.158; 0.027
Pre-event * Stability	-0.051	-0.151; 0.049	-0.024	-0.131; 0.081	-0.023	-0.126; 0.080
Post-event * Stability	0.165	0.069; 0.260	0.154	0.056; 0.252	0.181	0.081; 0.283

	**Without the United Kingdom** (*N* = 1,196)	
Predictors	b	95%-CI
Intercept	-0.111	-0.176; -0.043
Level	-0.137	-0.225; -0.049
Pre-event	-0.033	-0.174; 0.106
Post-event	0.134	0.038; 0.228
Stability	0.237	0.169; 0.305
Level * Stability	-0.027	-0.110; 0.056
Pre-event * Stability	-0.061	-0.192; 0.065
Post-event * Stability	0.193	0.094; 0.292

Notes. Displays fixed-effect coefficients of Bayesian multilevel models. The outcome of all models was well-being. b = unstandardized regression weight; 95%-CI = 95% credible interval around the estimate.

Supplementary Table 29. Coefficients of Model 2d including additional level-2 predictors in global data.

	**Model 2d**		**Model 2d + Stability**	
Predictors	b	95%-CI	b	95%-CI
Intercept	0.116	0.031; 0.202	-0.080	-0.160; 0.002
Level	-0.150	-0.212; -0.088	-0.137	-0.199; -0.074
Pre-event	-0.051	-0.141; 0.039	-0.034	-0.121; 0.054
Post-event	0.065	-0.015; 0.145	0.092	0.014; 0.169
Stability			0.315	0.260; 0.369
Level * Stability			-0.035	-0.095; 0.025
Pre-event * Stability			0.016	-0.074; 0.104
Post-event * Stability			0.073	-0.003; 0.150
European	-0.246	-0.328; -0.162	0.003	-0.075; 0.082

	**Model 2d + Age**		**Model 2d + Gender**	
Predictors	b	95%-CI	b	95%-CI
Intercept	0.074	-0.015; 0.163	0.090	-0.003; 0.181
Level	-0.150	-0.213; -0.087	-0.149	-0.220; -0.079
Pre-event	-0.032	-0.118; 0.055	-0.056	-0.158; 0.042
Post-event	0.075	-0.005; 0.155	0.061	-0.028; 0.151
SDV	0.028	-0.032; 0.089	0.127	-0.017; 0.266
Level * SDV	0.020	-0.040; 0.081	-0.003	-0.156; 0.149
Pre-event * SDV	-0.164	-0.247; -0.078	-0.053	-0.285; 0.177
Post-event * SDV	0.044	-0.038; 0.126	0.017	-0.184; 0.220
European	-0.196	-0.287; -0.106	-0.244	-0.327; -0.159

Supplementary Table 29. (continued)

	Model 2d + Political attitude		Model 2d + Social status	
Predictors	b	95%-CI	b	95%-CI
Intercept	0.107	0.022; 0.193	0.110	0.027; 0.196
Level	-0.153	-0.215; -0.091	-0.152	-0.214; -0.089
Pre-event	-0.052	-0.142; 0.037	-0.024	-0.116; 0.067
Post-event	0.064	-0.016; 0.144	0.085	0.005; 0.166
SDV	0.008	-0.048; 0.066	0.073	0.017; 0.130
Level * SDV	-0.067	-0.127; -0.007	0.010	-0.051; 0.072
Pre-event * SDV	-0.022	-0.117; 0.072	-0.065	-0.163; 0.032
Post-event * SDV	0.041	-0.041; 0.121	0.098	0.018; 0.178
European	-0.235	-0.318; -0.151	-0.239	-0.322; -0.155

Notes. N = 1,735. Displays fixed-effect coefficients of Bayesian multilevel models. The outcome of all models was well-being. b = unstandardized regression weight; 95%-CI = 95% credible interval around the estimate; SDV = sociodemographic variable as indicated in the column head.

Supplementary Table 30. Predicting well-being by the salience of the war and additional level-2 predictors in global data.

	**Salience**		**Salience (WS/BS)**	
Predictors	b	95%-CI	b	95%-CI
Intercept	0.191	0.118; 0.265	0.189	0.112; 0.266
Tweets	-0.050	-0.069; -0.030		
Tweets (WS)			-0.051	-0.070; -0.030
Tweets (BS)			-0.016	-0.086; 0.053
European	-0.249	-0.333; -0.166	-0.243	-0.331; -0.155

	**Salience + Stability**		**Salience (WS/BS) + Stability**	
Predictors	b	95%-CI	b	95%-CI
Intercept	-0.013	-0.082; 0.055	-0.002	-0.073; 0.069
Tweets	-0.057	-0.076; -0.037		
Tweets (WS)			-0.056	-0.077; -0.035
Tweets (BS)			-0.055	-0.119; 0.007
Stability	0.363	0.330; 0.396	0.364	0.332; 0.397
Tweets * Stability	0.014	-0.004; 0.032		
Tweets (WS) * Stability			0.016	-0.003; 0.035
Tweets (BS) * Stability			-0.007	-0.063; 0.050
European	0.015	-0.063; 0.094	0.003	-0.078; 0.085

	**Salience + Age**		**Salience (WS/BS) + Age**	
Predictors	b	95%-CI	b	95%-CI
Intercept	0.131	0.053; 0.209	0.120	0.039; 0.202
Tweets	-0.051	-0.071; -0.030		
Tweets (WS)			-0.051	-0.072; -0.030
Tweets (BS)			-0.027	-0.098; 0.045
SDV	0.090	0.053; 0.128	0.094	0.056; 0.132
Tweets * SDV	0.003	-0.016; 0.022		
Tweets (WS) * SDV			0.002	-0.018; 0.021
Tweets (BS) * SDV			0.036	-0.029; 0.100
European	-0.168	-0.258; -0.078	-0.155	-0.251; -0.059

Supplementary Table 30. (continued)

	Salience + Gender		Salience (WS/BS) + Gender	
Predictors	b	95%-CI	b	95%-CI
Intercept	0.162	0.085; 0.240	0.163	0.084; 0.244
Tweets	-0.050	-0.072; -0.028		
Tweets (WS)			-0.051	-0.073; -0.028
Tweets (BS)			-0.021	-0.101; 0.056
SDV	0.142	0.056; 0.230	0.144	0.057; 0.231
Tweets * SDV	-0.001	-0.049; 0.047		
Tweets (WS) * SDV			0.001	-0.049; 0.049
Tweets (BS) * SDV			-0.030	-0.193; 0.135
European	-0.246	-0.332; -0.162	-0.245	-0.333; -0.156

	Salience + Political attitude		Salience (WS/BS) + Political attitide	
Predictors	b	95%-CI	b	95%-CI
Intercept	0.184	0.109; 0.260	0.181	0.102; 0.258
Tweets	-0.051	-0.070; -0.031		
Tweets (WS)			-0.052	-0.072; -0.032
Tweets (BS)			-0.013	-0.082; 0.056
SDV	0.049	0.014; 0.084	0.052	0.017; 0.087
Tweets * SDV	-0.018	-0.037; 0.000		
Tweets (WS) * SDV			-0.018	-0.037; 0.001
Tweets (BS) * SDV			-0.019	-0.082; 0.044
European	-0.238	-0.325; -0.155	-0.233	-0.321; -0.142

Supplementary Table 30. (continued)

	Salience + Social status		Salience (WS/BS) + Social status	
Predictors	b	95%-CI	b	95%-CI
Intercept	0.184	0.112; 0.257	0.180	0.104; 0.253
Tweets	-0.049	-0.068; -0.029		
Tweets (WS)			-0.050	-0.070; -0.030
Tweets (BS)			-0.009	-0.079; 0.061
SDV	0.131	0.097; 0.166	0.131	0.096; 0.166
Tweets * SDV	-0.009	-0.028; 0.009		
Tweets (WS) * SDV			-0.010	-0.029; 0.010
Tweets (BS) * SDV			-0.002	-0.065; 0.061
European	-0.237	-0.319; -0.153	-0.229	-0.313; -0.142

Notes. N = 1,735. Displays fixed-effect coefficients of Bayesian multilevel models. The outcome of all models was well-being. WS = within-subjects component; BS = between-subjects component; b = unstandardized regression weight; 95%-CI = 95% credible interval around the estimate; SDV = sociodemographic variable as indicated in the column head.

Supplementary Table 31. Coefficients of Model 2d including additional level-2 predictors with shorter time frame.

	Model 2d		Model 2d + Stability	
Predictors	b	95%-CI	b	95%-CI
Intercept	-0.214	-0.341; -0.086	-0.228	-0.349; -0.106
Level	-0.355	-0.508; -0.201	-0.351	-0.504; -0.199
Pre-event	0.150	-0.652; 0.955	0.055	-0.744; 0.863
Post-event	1.114	0.263; 1.957	1.151	0.297; 2.004
Stability			0.300	0.179; 0.423
Level * Stability			-0.050	-0.200; 0.100
Pre-event * Stability			1.008	0.218; 1.789
Post-event * Stability			-0.258	-1.145; 0.625

Supplementary Table 31. (continued)

	Model 2d + Age		Model 2d + Gender	
Predictors	b	95%-CI	b	95%-CI
Intercept	-0.216	-0.342; -0.091	-0.197	-0.337; -0.056
Level	-0.357	-0.510; -0.205	-0.325	-0.497; -0.154
Pre-event	0.149	-0.658; 0.953	0.065	-0.822; 0.952
Post-event	1.122	0.277; 1.987	1.002	0.060; 1.941
SDV	0.065	-0.059; 0.190	-0.103	-0.440; 0.234
Level * SDV	0.082	-0.068; 0.233	-0.151	-0.546; 0.249
Pre-event * SDV	-0.327	-1.131; 0.480	0.415	-1.616; 2.462
Post-event * SDV	-0.329	-1.173; 0.523	0.810	-1.464; 3.069

	Model 2d + Political attitude		Model 2d + Social status	
Predictors	b	95%-CI	b	95%-CI
Intercept	-0.216	-0.343; -0.088	-0.211	-0.335; -0.087
Level	-0.358	-0.513; -0.204	-0.350	-0.502; -0.200
Pre-event	0.157	-0.655; 0.969	0.149	-0.654; 0.957
Post-event	1.138	0.267; 2.002	1.112	0.268; 1.962
SDV	-0.025	-0.157; 0.108	0.180	0.051; 0.309
Level * SDV	-0.064	-0.223; 0.098	0.042	-0.113; 0.197
Pre-event * SDV	-0.004	-0.848; 0.837	0.496	-0.319; 1.308
Post-event * SDV	0.279	-0.617; 1.168	-0.320	-1.185; 0.560

Notes. N = 341. Displays fixed-effect coefficients of Bayesian multilevel models. The outcome of all models was well-being. b = unstandardized regression weight; 95%-CI = 95% credible interval around the estimate; SDV = sociodemographic variable as indicated in the column head.

Supplementary Table 32. Predicting well-being by the salience of the war and additional level-2 predictors with shorter time frame.

	Salience		Salience (WS/BS)	
Predictors	b	95%-CI	b	95%-CI
Intercept	-0.006	-0.088; 0.077	-0.004	-0.088; 0.081
Tweets	-0.134	-0.183; -0.086		
Tweets (WS)			-0.133	-0.185; -0.082
Tweets (BS)			-0.143	-0.288; 0.002

Supplementary Table 32. (continued)

	Salience + Stability		Salience (WS/BS) + Stability	
Predictors	b	95%-CI	b	95%-CI
Intercept	-0.012	-0.088; 0.064	-0.011	-0.091; 0.068
Tweets	-0.137	-0.186; -0.089		
Tweets (WS)			-0.134	-0.186; -0.083
Tweets (BS)			-0.144	-0.280; -0.006
Stability	0.252	0.175; 0.329	0.263	0.182; 0.345
Tweets * Stability	0.021	-0.029; 0.069		
Tweets (WS) * Stability			0.015	-0.037; 0.066
Tweets (BS) * Stability			0.081	-0.062; 0.223

	Salience + Age		Salience (WS/BS) + Age	
Predictors	b	95%-CI	b	95%-CI
Intercept	-0.006	-0.088; 0.077	-0.005	-0.090; 0.079
Tweets	-0.135	-0.184; -0.086		
Tweets (WS)			-0.134	-0.185; -0.082
Tweets (BS)			-0.125	-0.274; 0.023
SDV	0.027	-0.055; 0.109	0.038	-0.048; 0.124
Tweets * SDV	0.013	-0.034; 0.061		
Tweets (WS) * SDV			0.006	-0.044; 0.056
Tweets (BS) * SDV			0.103	-0.069; 0.274

	Salience + Gender		Salience (WS/BS) + Gender	
Predictors	b	95%-CI	b	95%-CI
Intercept	-0.003	-0.093; 0.087	-0.002	-0.095; 0.092
Tweets	-0.128	-0.182; -0.074		
Tweets (WS)			-0.128	-0.185; -0.071
Tweets (BS)			-0.135	-0.293; 0.023
SDV	-0.012	-0.229; 0.200	-0.007	-0.235; 0.219
Tweets * SDV	-0.026	-0.151; 0.102		
Tweets (WS) * SDV			-0.030	-0.162; 0.103
Tweets (BS) * SDV			0.035	-0.373; 0.434

Supplementary Table 32. (continued)

	Salience + Political attitude		Salience (WS/BS) + Political attitude	
Predictors	b	95%-CI	b	95%-CI
Intercept	-0.006	-0.088; 0.077	0.002	-0.082; 0.090
Tweets	-0.135	-0.184; -0.086		
Tweets (WS)			-0.135	-0.187; -0.083
Tweets (BS)			-0.134	-0.278; 0.011
SDV	0.014	-0.071; 0.098	0.045	-0.046; 0.137
Tweets * SDV	-0.022	-0.073; 0.029		
Tweets (WS) * SDV			-0.037	-0.091; 0.015
Tweets (BS) * SDV			0.116	-0.037; 0.265

	Salience + Social status		Salience (WS/BS) + Social status	
Predictors	b	95%-CI	b	95%-CI
Intercept	-0.005	-0.086; 0.075	-0.002	-0.087; 0.083
Tweets	-0.133	-0.181; -0.085		
Tweets (WS)			-0.133	-0.184; -0.082
Tweets (BS)			-0.133	-0.278; 0.011
SDV	0.118	0.034; 0.199	0.117	0.029; 0.202
Tweets * SDV	0.041	-0.007; 0.090		
Tweets (WS) * SDV			0.040	-0.012; 0.092
Tweets (BS) * SDV			0.050	-0.103; 0.202

Notes. N = 341. Displays fixed-effect coefficients of Bayesian multilevel models. The outcome of all models was well-being. WS = within-subjects component; BS = between-subjects component; b = unstandardized regression weight; 95%-CI = 95% credible interval around the estimate; SDV = sociodemographic variable as indicated in the column head.

Supplementary Table 33. Coefficients of Model 2d including additional level-2 predictors with longer time frame.

	Model 2d		Model 2d + Stability	
Predictors	b	95%-CI	b	95%-CI
Intercept	-0.086	-0.139; -0.032	-0.063	-0.115; -0.012
Level	-0.143	-0.213; -0.072	-0.113	-0.185; -0.042
Pre-event	-0.081	-0.153; -0.011	-0.061	-0.129; 0.009
Post-event	0.084	0.035; 0.133	0.073	0.025; 0.120
SDV			0.292	0.239; 0.345
Level * SDV			0.003	-0.064; 0.070
Pre-event * SDV			-0.020	-0.086; 0.046
Post-event * SDV			0.071	0.023; 0.120

	Model 2d + Age		Model 2d + Gender	
Predictors	b	95%-CI	b	95%-CI
Intercept	-0.083	-0.137; -0.028	-0.125	-0.184; -0.064
Level	-0.136	-0.208; -0.063	-0.158	-0.235; -0.080
Pre-event	-0.048	-0.120; 0.024	-0.096	-0.172; -0.019
Post-event	0.088	0.039; 0.138	0.096	0.041; 0.150
SDV	0.044	-0.014; 0.102	0.191	0.056; 0.328
Level * SDV	-0.020	-0.089; 0.051	0.067	-0.118; 0.252
Pre-event * SDV	-0.046	-0.099; 0.007	0.050	-0.152; 0.252
Post-event * SDV	0.041	-0.011; 0.094	-0.042	-0.166; 0.082

	Model 2d + Political attitude		Model 2d + Social status	
Predictors	b	95%-CI	b	95%-CI
Intercept	-0.087	-0.141; -0.034	-0.076	-0.130; -0.022
Level	-0.146	-0.217; -0.075	-0.140	-0.211; -0.068
Pre-event	-0.082	-0.153; -0.011	-0.043	-0.118; 0.031
Post-event	0.085	0.036; 0.134	0.085	0.037; 0.134
SDV	0.050	-0.005; 0.105	0.138	0.085; 0.191
Level * SDV	-0.030	-0.104; 0.042	0.055	-0.014; 0.126
Pre-event * SDV	0.013	-0.065; 0.091	-0.044	-0.117; 0.030
Post-event * SDV	-0.011	-0.061; 0.039	0.008	-0.040; 0.057

Notes. N = 1,915. Displays fixed-effect coefficients of Bayesian multilevel models. The outcome of all models was well-being. b = unstandardized regression weight; 95%-CI = 95% credible interval around the estimate; SDV = sociodemographic variable as indicated in the column head.

Supplementary Table 34. Predicting well-being by the salience of the war and additional level-2 predictors with longer time frame.

	**Salience**		**Salience (WS/BS)**	
Predictors	b	95%-CI	b	95%-CI
Intercept	0.034	0.000; 0.067	0.038	0.005; 0.071
Tweets	-0.054	-0.077; -0.032		
Tweets (WS)			-0.031	-0.057; -0.005
Tweets (BS)			-0.185	-0.255; -0.116

	**Salience + Stability**		**Salience (WS/BS) + Stability**	
Predictors	b	95%-CI	b	95%-CI
Intercept	0.033	0.004; 0.063	0.035	0.006; 0.065
Tweets	-0.053	-0.075; -0.031		
Tweets (WS)			-0.034	-0.060; -0.008
Tweets (BS)			-0.149	-0.211; -0.087
Stability	0.352	0.322; 0.382	0.352	0.323; 0.381
Tweets * Stability	-0.021	-0.043; 0.000		
Tweets (WS) * Stability			-0.022	-0.049; 0.003
Tweets (BS) * Stability			-0.028	-0.089; 0.034

	**Salience + Age**		**Salience (WS/BS) + Age**	
Predictors	b	95%-CI	b	95%-CI
Intercept	0.035	0.003; 0.068	0.040	0.007; 0.072
Tweets	-0.052	-0.074; -0.029		
Tweets (WS)			-0.032	-0.057; -0.005
Tweets (BS)			-0.173	-0.242; -0.103
SDV	0.098	0.065; 0.132	0.100	0.067; 0.134
Tweets * SDV	-0.015	-0.038; 0.008		
Tweets (WS) * SDV			-0.022	-0.049; 0.004
Tweets (BS) * SDV			0.033	-0.037; 0.103

Supplementary Table 34. (continued)

	Salience + Gender		Salience (WS/BS) + Gender	
Predictors	b	95%-CI	b	95%-CI
Intercept	0.012	-0.026; 0.048	0.015	-0.021; 0.051
Tweets	-0.055	-0.080; -0.031		
Tweets (WS)			-0.029	-0.058; 0.000
Tweets (BS)			-0.205	-0.281; -0.127
SDV	0.130	0.043; 0.216	0.127	0.042; 0.211
Tweets * SDV	-0.001	-0.060; 0.058		
Tweets (WS) * SDV			-0.016	-0.082; 0.050
Tweets (BS) * SDV			0.084	-0.099; 0.271

	Salience + Political attitude		Salience (WS/BS) + Political attitude	
Predictors	b	95%-CI	b	95%-CI
Intercept	0.034	0.001; 0.067	0.038	0.005; 0.071
Tweets	-0.054	-0.077; -0.032		
Tweets (WS)			-0.031	-0.057; -0.005
Tweets (BS)			-0.183	-0.254; -0.113
SDV	0.040	0.006; 0.073	0.040	0.006; 0.073
Tweets * SDV	-0.002	-0.026; 0.022		
Tweets (WS) * SDV			-0.001	-0.028; 0.026
Tweets (BS) * SDV			-0.002	-0.080; 0.074

	Salience + Social status		Salience (WS/BS) + Social status	
Predictors	b	95%-CI	b	95%-CI
Intercept	0.036	0.004; 0.068	0.040	0.008; 0.073
Tweets	-0.053	-0.075; -0.031		
Tweets (WS)			-0.033	-0.058; -0.006
Tweets (BS)			-0.167	-0.236; -0.098
SDV	0.143	0.110; 0.176	0.139	0.106; 0.172
Tweets * SDV	0.001	-0.022; 0.023		
Tweets (WS) * SDV			-0.002	-0.028; 0.024
Tweets (BS) * SDV			0.029	-0.042; 0.100

Notes. N = 1,915. Displays fixed-effect coefficients of Bayesian multilevel models. The outcome of all models was well-being. WS = within-subjects component; BS = between-subjects component; b = unstandardized regression weight; 95%-CI = 95% credible interval around the estimate; SDV = sociodemographic variable as indicated in the column head.

Supplementary Table 35. Predicting time-lagged well-being by the salience of the war and additional level-2 predictors.

	**Salience**		**Salience (WS/BS)**	
Predictors	b	95%-CI	b	95%-CI
(Intercept)	-0.004	-0.044; 0.036	-0.010	-0.051; 0.031
Tweets	-0.044	-0.070; -0.018		
Tweets (WS)			-0.037	-0.064; -0.009
Tweets (BS)			-0.111	-0.206; -0.016

	**Salience + Stability**		**Salience (WS/BS) + Stability**	
Predictors	b	95%-CI	b	95%-CI
Intercept	-0.003	-0.040; 0.033	-0.007	-0.043; 0.029
Tweets	-0.044	-0.070; -0.018		
Tweets (WS)			-0.038	-0.066; -0.011
Tweets (BS)			-0.096	-0.183; -0.009
Stability	0.335	0.299; 0.371	0.333	0.296; 0.369
Tweets * Stability	-0.010	-0.035; 0.014		
Tweets (WS) * Stability			-0.007	-0.034; 0.019
Tweets (BS) * Stability			-0.042	-0.126; 0.043

	**Salience + Age**		**Salience (WS/BS) + Age**	
Predictors	b	95%-CI	b	95%-CI
Intercept	-0.001	-0.040; 0.039	-0.006	-0.047; 0.034
Tweets	-0.043	-0.068; -0.016		
Tweets (WS)			-0.037	-0.064; -0.008
Tweets (BS)			-0.102	-0.197; -0.008
SDV	0.083	0.041; 0.124	0.085	0.040; 0.129
Tweets * SDV	-0.007	-0.033; 0.019		
Tweets (WS) * SDV			-0.009	-0.038; 0.019
Tweets (BS) * SDV			0.014	-0.082; 0.114

Supplementary Table 35. (continued)

	**Salience + Gender**		**Salience (WS/BS) + Gender**	
Predictors	b	95%-CI	b	95%-CI
Intercept	-0.029	-0.073; 0.015	-0.036	-0.081; 0.009
Tweets	-0.044	-0.072; -0.015		
Tweets (WS)			-0.034	-0.064; -0.003
Tweets (BS)			-0.133	-0.238; -0.030
SDV	0.135	0.033; 0.238	0.142	0.039; 0.246
Tweets * SDV	-0.010	-0.077; 0.058		
Tweets (WS) * SDV			-0.022	-0.094; 0.050
Tweets (BS) * SDV			0.112	-0.149; 0.367

	**Salience + Political attitude**		**Salience (WS/BS) + Political attitude**	
Predictors	b	95%-CI	b	95%-CI
Intercept	-0.005	-0.045; 0.035	-0.008	-0.049; 0.033
Tweets	-0.044	-0.070; -0.018		
Tweets (WS)			-0.037	-0.065; -0.009
Tweets (BS)			-0.104	-0.199; -0.007
SDV	0.038	-0.002; 0.078	0.039	-0.003; 0.079
Tweets * SDV	-0.002	-0.029; 0.025		
Tweets (WS) * SDV			-0.003	-0.032; 0.026
Tweets (BS) * SDV			0.015	-0.086; 0.116

	**Salience + Social status**		**Salience WS BS + Social status**	
Predictors	b	95%-CI	b	95%-CI
Intercept	-0.001	-0.039; 0.038	-0.005	-0.045; 0.036
Tweets	-0.044	-0.070; -0.018		
Tweets (WS)			-0.038	-0.065; -0.010
Tweets (BS)			-0.101	-0.194; -0.008
SDV	0.150	0.110; 0.190	0.153	0.112; 0.195
Tweets * SDV	0.006	-0.020; 0.033		
Tweets (WS) * SDV			0.002	-0.027; 0.031
Tweets (BS) * SDV			0.056	-0.042; 0.156

Notes. N = 1,341. Displays fixed-effect coefficients of Bayesian multilevel models. The outcome of all models was the well-being on the consecutive day (lag-1). WS = within-subjects component; BS = between-subjects component; b = unstandardized regression weight; 95%-CI = 95% credible interval around the estimate; SDV = sociodemographic variable as indicated in the column head.

Supplementary Table 36. Predicting well-being and its sub-facets by Stability and its sub-facets.

	**Negative affect**		**Positive affect**		**Well-being**	
Predictors	b	95%-CI	b	95%-CI	b	95%-CI
Intercept	0.064	-0.005; 0.131	-0.081	-0.144; -0.017	-0.090	-0.155; -0.026
Level	0.178	0.093; 0.262	-0.118	-0.193; -0.045	-0.183	-0.263; -0.103
Pre-event	0.009	-0.093; 0.109	0.070	-0.046; 0.183	0.041	-0.063; 0.144
Post-event	-0.067	-0.162; 0.028	0.129	0.036; 0.223	0.122	0.031; 0.213
Stability	-0.218	-0.286; -0.149	0.202	0.138; 0.267	0.245	0.180; 0.311
Level * Stability	-0.003	-0.080; 0.075	-0.039	-0.109; 0.031	-0.026	-0.103; 0.049
Pre-event * Stability	0.059	-0.036; 0.155	-0.019	-0.128; 0.088	-0.042	-0.141; 0.056
Post-event * Stability	-0.123	-0.220; -0.026	0.146	0.050; 0.242	0.160	0.065; 0.254

	**Negative affect**		**Positive affect**		**Well-being**	
Predictors	b	95%-CI	b	95%-CI	b	95%-CI
Intercept	0.064	-0.004; 0.133	-0.084	-0.147; -0.020	-0.093	-0.157; -0.029
Level	0.168	0.081; 0.253	-0.124	-0.199; -0.050	-0.183	-0.262; -0.103
Pre-event	-0.001	-0.102; 0.098	0.086	-0.029; 0.199	0.056	-0.047; 0.157
Post-event	-0.086	-0.182; 0.008	0.149	0.055; 0.244	0.147	0.055; 0.238
Neuroticism	0.210	0.140; 0.278	-0.232	-0.297; -0.168	-0.259	-0.324; -0.193
Level * Neuroticism	-0.012	-0.093; 0.069	-0.007	-0.076; 0.065	0.005	-0.072; 0.083
Pre-event * Neuroticism	-0.087	-0.181; 0.006	0.044	-0.061; 0.149	0.075	-0.021; 0.173
Post-event * Neuroticism	0.151	0.053; 0.248	-0.122	-0.219; -0.025	-0.162	-0.258; -0.067

Supplementary Table 36. (continued)

	**Negative affect**		**Positive affect**		**Well-being**	
Predictors	b	95%-CI	b	95%-CI	b	95%-CI
Intercept	0.069	0.000; 0.138	-0.080	-0.145; -0.014	-0.092	-0.158; -0.026
Level	0.201	0.117; 0.282	-0.126	-0.201; -0.052	-0.203	-0.282; -0.123
Pre-event	0.042	-0.057; 0.141	0.041	-0.078; 0.156	0.010	-0.095; 0.113
Post-event	-0.044	-0.139; 0.050	0.100	0.006; 0.196	0.091	-0.004; 0.186
Concientiousness	-0.184	-0.254; -0.114	0.087	0.021; 0.153	0.160	0.093; 0.228
Level * Conscientiousness	-0.068	-0.147; 0.011	-0.041	-0.113; 0.030	0.010	-0.067; 0.088
Pre-event * Conscientiousness	0.046	-0.046; 0.139	-0.017	-0.122; 0.089	-0.030	-0.125; 0.065
Post-event * Conscientiousness	0.043	-0.057; 0.142	0.049	-0.049; 0.147	0.003	-0.095; 0.100

	**Negative affect**		**Positive affect**		**Well-being**	
Predictors	b	95%-CI	b	95%-CI	b	95%-CI
Intercept	0.064	-0.005; 0.133	-0.080	-0.145; -0.014	-0.088	-0.155; -0.022
Level	0.183	0.099; 0.267	-0.119	-0.193; -0.044	-0.188	-0.267; -0.108
Pre-event	0.056	-0.045; 0.157	0.040	-0.076; 0.154	0.003	-0.104; 0.105
Post-event	-0.045	-0.142; 0.050	0.109	0.013; 0.204	0.096	0.003; 0.189
Agreeableness	-0.053	-0.123; 0.017	0.095	0.029; 0.162	0.086	0.018; 0.154
Level * Agreeableness	0.072	-0.010; 0.156	-0.085	-0.160; -0.011	-0.099	-0.180; -0.020
Pre-event * Agreeableness	-0.066	-0.165; 0.035	0.070	-0.044; 0.182	0.088	-0.016; 0.189
Post-event * Agreeableness	-0.146	-0.243; -0.049	0.135	0.039; 0.232	0.167	0.072; 0.263

Notes. N = 1,341. Displays fixed-effect coefficients of Bayesian multilevel models. The outcome of each model is indicated in the column head. b = unstandardized regression weight; 95%-CI = 95% credible interval around the estimate.

Supplementary Table 37. Predicting well-being and its sub-facets by Plasticity and its sub-facets.

	**Negative affect**		**Positive affect**		**Well-being**	
Predictors	b	95%-CI	b	95%-CI	b	95%-CI
Intercept	0.067	-0.002; 0.136	-0.082	-0.147; -0.018	-0.092	-0.159; -0.026
Level	0.195	0.112; 0.277	-0.132	-0.208; -0.058	-0.204	-0.283; -0.124
Pre-event	0.053	-0.047; 0.153	0.039	-0.077; 0.154	0.004	-0.101; 0.107
Post-event	-0.044	-0.139; 0.052	0.107	0.012; 0.202	0.094	0.001; 0.187
Plasticity	-0.067	-0.137; 0.003	0.141	0.075; 0.207	0.125	0.058; 0.192
Level * Plasticity	0.020	-0.059; 0.099	-0.038	-0.106; 0.031	-0.031	-0.107; 0.044
Pre-event * Plasticity	0.037	-0.056; 0.131	0.006	-0.099; 0.111	-0.016	-0.112; 0.080
Post-event * Plasticity	-0.010	-0.108; 0.089	0.026	-0.072; 0.124	0.018	-0.079; 0.115

	**Negative affect**		**Positive affect**		**Well-being**	
Predictors	b	95%-CI	b	95%-CI	b	95%-CI
Intercept	0.067	-0.002; 0.135	-0.082	-0.147; -0.019	-0.092	-0.158; -0.027
Level	0.194	0.111; 0.275	-0.132	-0.207; -0.057	-0.203	-0.282; -0.123
Pre-event	0.049	-0.050; 0.149	0.041	-0.076; 0.155	0.007	-0.098; 0.111
Post-event	-0.046	-0.142; 0.051	0.108	0.015; 0.205	0.098	0.003; 0.191
Extraversion	-0.129	-0.199; -0.059	0.199	0.135; 0.264	0.197	0.131; 0.262
Level * Extraversion	-0.011	-0.085; 0.064	0.009	-0.058; 0.076	0.016	-0.056; 0.088
Pre-event * Extraversion	0.004	-0.085; 0.092	0.010	-0.091; 0.113	0.005	-0.087; 0.095
Post-event * Extraversion	-0.003	-0.103; 0.097	0.020	-0.079; 0.119	0.008	-0.089; 0.106

	**Negative affect**		**Positive affect**		**Well-being**	
Predictors	b	95%-CI	b	95%-CI	b	95%-CI
Intercept	0.065	-0.004; 0.134	-0.079	-0.144; -0.014	-0.089	-0.155; -0.022
Level	0.194	0.112; 0.276	-0.127	-0.201; -0.053	-0.202	-0.281; -0.123
Pre-event	0.055	-0.044; 0.154	0.038	-0.078; 0.153	0.004	-0.101; 0.107
Post-event	-0.040	-0.136; 0.057	0.101	0.005; 0.196	0.088	-0.006; 0.182
Openness	0.034	-0.036; 0.103	0.012	-0.053; 0.079	-0.011	-0.078; 0.055
Level * Openness	0.048	-0.036; 0.131	-0.087	-0.161; -0.012	-0.076	-0.157; 0.003
Pre-event * Openness	0.055	-0.044; 0.153	0.014	-0.098; 0.127	-0.023	-0.125; 0.080
Post-event * Openness	-0.025	-0.120; 0.070	0.038	-0.057; 0.133	0.036	-0.057; 0.129

Notes. N = 1,341. Displays fixed-effect coefficients of Bayesian multilevel models. The outcome of each model is indicated in the column head. b = unstandardized regression weight; 95%-CI = 95% credible interval around the estimate.

Supplementary Table 38. Coefficients and model fits of models representing different societal well-being trajectories.

	Model 1a		Model 1b		Model 1c	
Predictors	b	95%-CI	b	95%-CI	b	95%-CI
Intercept	0.018	-0.030; 0.065	0.050	-0.002; 0.102	-0.046	-0.104; 0.012
Level			0.127	0.056; 0.199		
Post-event					0.151	0.065; 0.238

	Model 1d		Model 2a		Model 2b	
Predictors	b	95%-CI	b	95%-CI	b	95%-CI
Intercept	-0.021	-0.087; 0.045	-0.020	-0.073; 0.032	-0.010	-0.070; 0.049
Time			0.124	0.060; 0.188	0.110	0.037; 0.181
Level	0.089	0.018; 0.160			0.040	-0.044; 0.126
Post-event	0.137	0.045; 0.226				

	Model 2c		Model 2d	
Predictors	b	95%-CI	b	95%-CI
Intercept	-0.020	-0.084; 0.044	-0.009	-0.076; 0.057
Level			0.050	-0.030; 0.130
Pre-event	0.160	0.050; 0.270	0.114	-0.008; 0.236
Post-event	0.135	0.045; 0.226	0.126	0.034; 0.216

Notes. N = 1,280. Displays fixed-effect coefficients of Bayesian multilevel models. The outcome of all models was societal well-being. b = unstandardized regression weight; 95%-CI = 95% credible interval around the estimate.

Supplementary Table 39. Coefficients of Model 2d including additional level-2 variables predicting societal well-being.

	Model 2d		Model 2d + Stability	
Predictors	b	95%-CI	b	95%-CI
Intercept	-0.009	-0.076; 0.057	-0.006	-0.072; 0.060
Level	0.050	-0.030; 0.130	0.079	-0.002; 0.161
Pre-event	0.114	-0.008; 0.236	0.142	0.019; 0.266
Post-event	0.126	0.034; 0.216	0.133	0.042; 0.224
Stability			0.100	0.032; 0.167
Level * Stability			-0.066	-0.139; 0.005
Pre-event * Stability			-0.059	-0.171; 0.054
Post-event * Stability			0.060	-0.034; 0.153

Supplementary Table 39. (continued)

	Model 2d + Age		Model 2d + Gender	
Predictors	b	95%-CI	b	95%-CI
Intercept	-0.021	-0.088; 0.046	0.023	-0.052; 0.097
Level	0.035	-0.046; 0.116	0.039	-0.048; 0.127
Pre-event	0.108	-0.016; 0.231	0.137	0.006; 0.270
Post-event	0.135	0.043; 0.228	0.153	0.053; 0.255
SDV	-0.133	-0.206; -0.061	-0.145	-0.315; 0.027
Level * SDV	0.042	-0.034; 0.119	0.076	-0.116; 0.273
Pre-event * SDV	-0.159	-0.275; -0.043	-0.112	-0.433; 0.209
Post-event * SDV	0.127	0.029; 0.225	-0.170	-0.404; 0.062

	Model 2d + Political attitude		Model 2d + Social status	
Predictors	b	95%-CI	b	95%-CI
Intercept	-0.009	-0.077; 0.057	-0.009	-0.076; 0.058
Level	0.045	-0.034; 0.125	0.054	-0.025; 0.135
Pre-event	0.118	-0.002; 0.240	0.144	0.020; 0.267
Post-event	0.125	0.035; 0.217	0.126	0.035; 0.219
SDV	0.028	-0.040; 0.097	0.047	-0.022; 0.114
Level * SDV	-0.035	-0.112; 0.042	-0.003	-0.078; 0.072
Pre-event * SDV	-0.103	-0.223; 0.016	-0.096	-0.219; 0.024
Post-event * SDV	-0.006	-0.099; 0.087	-0.024	-0.117; 0.069

Notes. N = 1,280. Displays fixed-effect coefficients of Bayesian multilevel models. The outcome of all models was societal well-being. b = unstandardized regression weight; 95%-CI = 95% credible interval around the estimate; SDV = sociodemographic variable as indicated in the column head.

Supplementary Table 40. Predicting societal well-being by the salience of the war and additional level-2 predictors.

	Salience		Salience (WS/BS)	
Predictors	b	95%-CI	b	95%-CI
Intercept	0.016	-0.032; 0.062	0.015	-0.032; 0.064
Tweets	-0.020	-0.045; 0.004		
Tweets (WS)			-0.024	-0.051; 0.001
Tweets (BS)			-0.008	-0.120; 0.100

Supplementary Table 40. (continued)

	**Salience + Stability**		**Salience (WS/BS) + Stability**	
Predictors	b	95%-CI	b	95%-CI
Intercept	0.015	-0.033; 0.060	0.016	-0.030; 0.063
Tweets	-0.017	-0.042; 0.008		
Tweets (WS)			-0.023	-0.051; 0.004
Tweets (BS)			-0.001	-0.107; 0.110
Stability	0.146	0.100; 0.193	0.150	0.102; 0.199
Tweets * Stability	-0.014	-0.037; 0.008		
Tweets (WS) * Stability			-0.013	-0.037; 0.010
Tweets (BS) * Stability			0.016	-0.091; 0.123

	**Salience + Age**		**Salience (WS/BS) + Age**	
Predictors	b	95%-CI	b	95%-CI
Intercept	0.015	-0.031; 0.061	0.015	-0.032; 0.062
Tweets	-0.021	-0.046; 0.004		
Tweets (WS)			-0.024	-0.051; 0.001
Tweets (BS)			-0.017	-0.129; 0.096
SDV	-0.071	-0.117; -0.024	-0.075	-0.127; -0.024
Tweets * SDV	-0.000	-0.024; 0.023		
Tweets (WS) * SDV			0.003	-0.022; 0.028
Tweets (BS) * SDV			-0.030	-0.140; 0.078

	**Salience + Gender**		**Salience (WS/BS) + Gender**	
Predictors	b	95%-CI	b	95%-CI
Intercept	0.061	0.009; 0.111	0.058	0.006; 0.111
Tweets	-0.026	-0.054; 0.001		
Tweets (WS)			-0.029	-0.059; -0.001
Tweets (BS)			-0.028	-0.147; 0.091
SDV	-0.230	-0.350; -0.106	-0.221	-0.344; -0.094
Tweets * SDV	0.031	-0.030; 0.093		
Tweets (WS) * SDV			0.028	-0.036; 0.090
Tweets (BS) * SDV			0.141	-0.185; 0.461

Supplementary Table 40. (continued)

	**Salience + Political attitude**		**Salience (WS/BS) + Political attitude**	
Predictors	b	95%-CI	b	95%-CI
Intercept	0.016	-0.030; 0.063	0.018	-0.029; 0.066
Tweets	-0.021	-0.046; 0.004		
Tweets (WS)			-0.025	-0.052; 0.001
Tweets (BS)			0.000	-0.112; 0.110
SDV	0.052	0.005; 0.099	0.056	0.007; 0.104
Tweets * SDV	-0.007	-0.032; 0.018		
Tweets (WS) * SDV			-0.007	-0.033; 0.019
Tweets (BS) * SDV			0.014	-0.104; 0.129

	**Salience + Social status**		**Salience (WS/BS) + Social status**	
Predictors	b	95%-CI	b	95%-CI
Intercept	0.018	-0.031; 0.064	0.019	-0.030; 0.067
Tweets	-0.021	-0.046; 0.004		
Tweets (WS)			-0.025	-0.053; 0.001
Tweets (BS)			-0.005	-0.115; 0.108
SDV	0.036	-0.011; 0.083	0.037	-0.012; 0.085
Tweets * SDV	0.001	-0.022; 0.026		
Tweets (WS) * SDV			0.003	-0.022; 0.028
Tweets (BS) * SDV			-0.004	-0.118; 0.109

Notes. N = 1,280. Displays fixed-effect coefficients of Bayesian multilevel models. The outcome of all models was societal well-being. WS = within-subjects component; BS = between-subjects component; b = unstandardized regression weight; 95%-CI = 95% credible interval around the estimate; SDV = sociodemographic variable as indicated in the column head.
