# Supplementary material for: Psychological well-being in Europe after the outbreak of war in Ukraine
Source: Nat Commun. 2024 Feb 20;15:1202. doi: 10.1038/s41467-024-44693-6 (PMC10879508; doi:10.1038/s41467-024-44693-6)
Supplement: Supplementary file 2 — Reporting Summary [file 41467_2024_44693_MOESM2_ESM.pdf]

nature portfolio

Corresponding author(s): Julian Scharbert

Last updated by author(s): Dec 12, 2023

Reporting Summary

Nature Portfolio wishes to improve the reproducibility of the work that we publish. This form provides structure for consistency and transparency in reporting. For further information on Nature Portfolio policies, see our Editorial Policies and the Editorial Policy Checklist.

Statistics

For all statistical analyses, confirm that the following items are present in the figure legend, table legend, main text, or Methods section.

n/a	Confirmed	
☐	☒	The exact sample size (n) for each experimental group/condition, given as a discrete number and unit of measurement
☐	☒	A statement on whether measurements were taken from distinct samples or whether the same sample was measured repeatedly
☐	☒	The statistical test(s) used AND whether they are one- or two-sided<br>*Only common tests should be described solely by name; describe more complex techniques in the Methods section.*
☐	☒	A description of all covariates tested
☐	☒	A description of any assumptions or corrections, such as tests of normality and adjustment for multiple comparisons
☐	☒	A full description of the statistical parameters including central tendency (e.g. means) or other basic estimates (e.g. regression coefficient) AND variation (e.g. standard deviation) or associated estimates of uncertainty (e.g. confidence intervals)
☐	☒	For null hypothesis testing, the test statistic (e.g. F, t, r) with confidence intervals, effect sizes, degrees of freedom and P value noted<br>*Give P values as exact values whenever suitable.*
☐	☒	For Bayesian analysis, information on the choice of priors and Markov chain Monte Carlo settings
☐	☒	For hierarchical and complex designs, identification of the appropriate level for tests and full reporting of outcomes
☒	☐	Estimates of effect sizes (e.g. Cohen's d, Pearson's r), indicating how they were calculated

Our web collection on statistics for biologists contains articles on many of the points above.

Software and code

Policy information about availability of computer code

Data collection	All data were collected using the survey software formr version v0.18.3.
Data analysis	All analyses were conducted using the statistical software R version 4.2.1.; the respective analysis scripts can be found at doi.org/10.17605/OSF.IO/8F3YU.

For manuscripts utilizing custom algorithms or software that are central to the research but not yet described in published literature, software must be made available to editors and reviewers. We strongly encourage code deposition in a community repository (e.g. GitHub). See the Nature Portfolio guidelines for submitting code & software for further information.

Data

Policy information about availability of data

All manuscripts must include a data availability statement. This statement should provide the following information, where applicable:

- Accession codes, unique identifiers, or web links for publicly available datasets
- A description of any restrictions on data availability
- For clinical datasets or third party data, please ensure that the statement adheres to our policy

The raw and processed data that support the findings of this study are publicly available on the OSF (doi.org/10.17605/OSF.IO/8F3YU). The data collection was part of the "Coping with Corona" project, which includes additional variables that we did not consider in this manuscript.

Research involving human participants, their data, or biological material

Policy information about studies with human participants or human data. See also policy information about sex, gender (identity/presentation), and sexual orientation and race, ethnicity and racism.

Reporting on sex and gender	Participants indicated their gender in the pre-survey by identifying themselves as female, male, or specifying another gender. Gender was considered as a predictor and moderator in the analyses conducted and these results are reported in the supplement. For these analyses, we only considered male and female participants because only 10 participants specified another gender, and these participants were most likely not representative of individuals with the same gender.
Reporting on race, ethnicity, or other socially relevant groupings	We do not report on race, ethnicity, or other socially relevant groupings.
Population characteristics	See section "Research sample" below.
Recruitment	See section "Sampling strategy" below.
Ethics oversight	The institutional review board from the University of Münster approved the study (2020-54-MB).

Note that full information on the approval of the study protocol must also be provided in the manuscript.

Field-specific reporting

Please select the one below that is the best fit for your research. If you are not sure, read the appropriate sections before making your selection.

☐ Life sciences ☒ Behavioural & social sciences ☐ Ecological, evolutionary & environmental sciences

For a reference copy of the document with all sections, see nature.com/documents/nr-reporting-summary-flat.pdf

Behavioural & social sciences study design

All studies must disclose on these points even when the disclosure is negative.

Study description	The study is a longitudinal, quantitative study applying the experience-sampling method.
Research sample	The study included participants from 17 European countries for the main analyses (43 countries for the supplementary analyses including the global sample). We focused on European countries because the outbreak of war has had the most direct consequences and was likely to be monitored closely by the public in these countries. The sample was not intentionally limited to a specific subgroup (e.g., university students) but is tilted towards younger (mean age = 25.7 years) and more female participants (80.5% female participants) and, thus, is not representative. Only individuals aged 18 years or older were allowed to participate in the study. Other than that, no restrictions for participating in the study were imposed (e.g., we did not deny participation for individuals with past or current diagnoses).
Sampling strategy	Participants were recruited using a convenience sampling strategy with the help of more than 40 collaborators worldwide as part of a global project aimed at investigating the psychological consequences of the COVID-19 pandemic. All researchers involved in the project disseminated a link to the online survey through various channels including social media, local and digital blackboards, mailing lists, university classes, recruitment panels, and local press releases in their respective countries. Self-selection biases might have occurred this way, as it is possible that primarily individuals who were interested in mental health and/or the COVID-19 pandemic chose to participate in the study.<br><br>Because we did not collect the data with the analyses conducted here in mind, we did not conduct any a priori sample-size calculation but included all data available in the period investigated here (considering the data exclusion criteria described below). However, the resulting sample size for the main analyses (N = 1,341, total assessments = 44,894) is sufficient to detect even small effects.
Data collection	Data were collected online with the data collection software formr. The core team of the project developed the initial survey in English and German. The English version was then translated into the languages of the included countries by professional translators. The collaborators who were native speakers in the respective languages revised the translations to ensure that the content of the survey was identical to the English version. One survey with a unique URL was set up for each language, respectively. Participants could choose their preferred language at the beginning of the survey and were redirected to the survey in the respective language.<br><br>After completing the pre-questionnaire, participants indicated their email address. We used this email address to send invitations to the short surveys over the following four weeks and the post-questionnaire. After receiving the invitation email, participants had 45 minutes to fill out the short survey. If participants did not respond within 20 minutes after receiving the invitation via email, we send out a reminder email.<br><br>Given that this study represents a post-hoc analysis of already collected data, the researchers were not aware of this study's hypotheses when collecting the data.
Timing	We restricted our dataset to the period of one month (31 days) before and after the Russian invasion (i.e., on the 63 days from

	January 24 to March 27, 2022).
Data exclusions	As preregistered, we excluded a) participants who indicated on the last page of the post-questionnaire that they had not answered the question in the survey conscientiously, b) participants who completed the pre-questionnaire too quickly (who took less than two seconds per item on average) and c) participants who provided state data on less than two days (this was minimally required to have variance on the change parameters). While criteria a) and b) were applied to the overall dataset of the project, criterion c) was specific to this study. This way, a) 21 and b) 36 participants were excluded from the overall sample and c) 282 participants were excluded from the subsample investigated here.
Non-participation	From 112 possible observations (4 randomly timed surveys on 28 days), participants provided 33.5 observations on average, corresponding to a response rate of 30%.
Randomization	Randomization does not apply in this study because we investigated a global event.

Reporting for specific materials, systems and methods

We require information from authors about some types of materials, experimental systems and methods used in many studies. Here, indicate whether each material, system or method listed is relevant to your study. If you are not sure if a list item applies to your research, read the appropriate section before selecting a response.

Materials & experimental systems

n/a	Involved in the study
☒	☐ Antibodies
☒	☐ Eukaryotic cell lines
☒	☐ Palaeontology and archaeology
☒	☐ Animals and other organisms
☒	☐ Clinical data
☒	☐ Dual use research of concern
☒	☐ Plants

Methods

n/a	Involved in the study
☒	☐ ChIP-seq
☒	☐ Flow cytometry
☒	☐ MRI-based neuroimaging
